# Cleavage-defective Topoisomerase I mutants sharply increase G-quadruplex-associated genomic instability

**DOI:** 10.15698/mic2022.03.771

**Published:** 2022-01-31

**Authors:** Alexandra Berroyer, Albino Bacolla, John A. Tainer, Nayun Kim

**Affiliations:** 1 Department of Microbiology and Molecular Genetics, University of Texas Health Science Center at Houston, Houston, TX 77030, USA.; 2 MD Anderson Cancer Center UT Health Graduate School of Biomedical Sciences, Houston, TX, 77030, USA.; 3 Department of Molecular and Cellular Oncology, University of Texas M.D. Anderson Cancer Center, Houston, TX 77030, USA.; 4 Department of Cancer Biology, University of Texas M.D. Anderson Cancer Center, Houston, TX 77030, USA.

**Keywords:** G-quadruplex, Topoisomerase, transcription, recombination, genome instability

## Abstract

Topoisomerase 1 (Top1) removes transcription-associated helical stress to suppress G4-formation and its induced recombination at genomic loci containing guanine-run containing sequences. Interestingly, Top1 binds tightly to G4 structures, and its inhibition or depletion can cause elevated instability at these genomic loci. Top1 is targeted by the widely used anti-cancer chemotherapeutic camptothecin (CPT) and its derivatives, which stabilize Top1 covalently attached on a DNA nick and prevent the re-ligation step. Here we investigated how CPT-resistance conferring Top1 mutants, which emerge in cancer patients and cells treated with CPT, affect G4-induced genomic instability in *S. cerevisiae*. We found that Top1 mutants form stable complexes with G4 DNA and that expression of Top1 cleavage-defective mutants but not a DNA-binding-defective mutant lead to significantly elevated instability at a G4-forming genomic locus. Elevated recombination rates were partly suppressed by their proteolytic removal by SPRTN homolog Wss1 SUMO-dependent metalloprotease *in vivo*. Furthermore, interaction between G4-DNA binding protein Nsr1, a homolog to clinically-relevant human nucleolin, and Top1 mutants lead to a synergistic increase in G4-associated recombination. These results in the yeast system are strengthened by our cancer genome data analyses showing that functionally detrimental mutations in Top1 correlate with an enrichment of mutations at G4 motifs. Our collective experimental and computational findings point to cooperative binding of Top1 cleavage-defective mutants and Nsr1 as promoting DNA replication blockage and exacerbating genomic instability at G4-motifs, thus complicating patient treatment.

## INTRODUCTION

Repetitive DNA sequences are hotspots of genomic instability [1]. The potential of these repetitive sequences to form non-B DNA structures that perturb replication and transcription exacerbates instability associated with them. For sequences containing guanine runs, non-B secondary DNA structures called G-quadruplexes (G4s) can arise when planar guanine tetrads made up of four guanine bases stabilized through Hoogsteen bonding stack upon one another [2, 3]. G4-formation usually requires the minimal sequence of GGGN_1-7_GGGN_1-7_GGGN_1-7_GGG, where N can be any of the DNA bases and comprises loops that extrude outward from the stacked guanine tetrads. While bioinformatics predicts >375,000 canonical G4-motifs in the human genome [4] and ~1,400 canonical G4-motifs in the *Saccharomyces cerevisiae* genome [5], more recent functional mapping of G4 DNA through a modified sequencing method identified 705,580 and 143 G4 DNAs in human and yeast genomes, respectively [6]. G4-motifs are enriched at functional genetic elements and regions such as ribosomal DNA, telomeric DNA, promoter regions, transcription start sites, sites of mitotic and meiotic recombination, and G-rich micro- and mini-satellites [5–8]. The non-random genomic locations of G4-motifs indicate that these structures have roles in the regulation of important cellular processes like transcription and programmed recombination.

While G4s have been reported to be *cis*-acting transcriptional regulatory elements, increasing evidence supports that dysregulation of G4-formation and/or resolution causes genomic instability and contributes to disease development and progression [3, 9, 10]. G4-motifs are found at many oncogenic translocation breakpoints in humans, implicating G4s in instigation of these cancer-associated genomic instability events [11–15]. Furthermore, “long G4-capable regions” in the human genome, or genomic regions harboring a density of at least 80 GGG repeats/kb, contain significantly more single nucleotide polymorphisms, insertions, and deletions than the proximally located non-G4 loci [16]. That is, G4-capable loci are hotspots of small-scale genome changes in addition to promoting large-scale genomic rearrangements, i.e. translocations. In *S. cerevisiae* and *Schizosaccharomyces pombe*, chromatin immunoprecipitation-next generation sequencing (ChIP-seq) approaches identified genomic G4 motifs as sites of increased replication fork pausing and DNA damage in the absence of DNA helicases Pif1 or Pfh1, respectively [17, 18]. More recent work in *S. cerevisiae* showed a slowing of the replication fork past a single defined G4 motif-containing genomic locus [19]. Since G4-formation and -stabilization can have such detrimental effects on genomic stability, regulation of these structures is critical for proper cell functioning.

Helical torsion is an important determinant of the formation and stability of secondary DNA structures including G4 DNA [20]. During the transcription of G-rich repetitive loci, negative supercoils accumulate behind RNA polymerase complexes and promote transiently single-stranded DNA patches that foster interaction among guanine bases [21, 22]. In yeast, elevated recombination and gross chromosomal rearrangements (GCRs) initiating at a model G4-motif from the mouse immunoglobulin switch region μ are dependent upon transcription [23, 24]. ChIP-seq studies utilizing the G4-specific antibody BG4 demonstrated that G4s form in nucleosome-depleted genomic regions in human cells [25]. Supporting the correlation between G4-formation and transcription, ChIP-seq data demonstrated that a greater number of G4 structures is associated with the highly transcribed genomes of cancer cells; around ~10,000 G4s were identified in the genomes of immortalized keratinocytes, while only ~1,000 G4s were identified in non-immortalized, primary keratinocytes.

Topoisomerase 1 (Top1) is an enzyme that relieves helical stress accumulated during transcription. Top1 binds duplex DNA, nicking one-strand of DNA with a catalytic tyrosine residue, and re-ligates the nick in DNA after controlled strand swiveling [26]. The complete deletion of theTop1-encoding gene in yeast or the siRNA-mediated knockdown of Top1 in mouse lymphoma B-cells results in highly elevated instability at genomic loci containing actively transcribed G4 DNA-forming sequences [23, 24, 27]. Further examination in yeast demonstrated that removal of transcription-associated negative excess helical tension by Top1 suppresses G4-induced recombination at these loci, indicating that Top1 plays an important role in protecting the genome by preventing G4-formation [28]. Top1 is the sole target of the widely used anti-cancer chemotherapeutic camptothecin (CPT) and its derivatives including irinotecan and topotecan (reviewed in [26]). CPT targets Top1 by stabilizing the Top1-cleavage complex (Top1cc) consisting of Top1 covalently attached to the 3' end of a DNA nick and then by preventing the re-ligation step. CPT cytotoxicity is mainly dependent on nuclear influx of CPT and DNA cleavage by Top1. While there are multiple ways in which cancer cells can become resistant to CPT, a prevalent mechanism of resistance is the mutation of Top1 such that the enzyme can no longer bind or cleave DNA. In fact, mutations that reduce Top1 DNA binding and/or cleavage are documented in cancer patients and cells treated with CPT or CPT-derivatives (reviewed in [29]). Whether these Top1 mutants affect G4-induced genomic instability has not been examined.

Interestingly, Top1 itself can tightly bind to G4 structures and promote formation of intermolecular G4s *in vitro* [30, 31]. The yeast Top1 catalytic mutant Top1Y727F, which can bind but not cleave duplex DNA, also binds to G4 DNA-forming oligos *in vitro* [29]. Expression of Top1Y727F in yeast results in extremely elevated instability at a model G4-motif that is significantly higher than *top1* null mutation, possibly resulting from the high-affinity binding of Top1Y727F to co-transcriptionally formed G4s *in vivo* [28]. Here, we expressed three distinct CPT-resistance conferring Top1 mutants in *S. cerevisiae* and found that the expression of Top1 mutants with a cleavage defect but not a mutant with a duplex DNA-binding defect led to significantly enhanced instability at a G4-forming genomic locus. Further experimentation revealed that Top1 cleavage-defective mutants bind G4 DNA structures *in vitro* and can be proteolytically removed by DNA-protein crosslink repair pathway *in vivo*. We also found that Nsr1, another G4-DNA binding protein and homolog to the human protein nucleolin [19], physically interacts with Top1 mutants and that expression of Top1 catalytic mutants and Nsr1 together has a synergistic effect on G4-induced recombination. We furthermore show that in cancer genomes, functionally detrimental mutations in Top1 correlate with enrichment of mutations at G4 motifs. Overall, our data suggest that Top1 cleavage-defective mutants and Nsr1 interact and bind to G4s cooperatively, which could result in DNA replication blockage and exacerbated genomic instability at G4-motifs.

## RESULTS

### Top1 mutants used in the study

Previously, we reported that the expression of catalytically dead Top1Y727F mutation results in a significant elevation of G4-associated genome instability over Top1-absence, indicating a gain of function by this catalytic mutant protein [28]. In order to determine the mechanism underlying such elevated G4-specific genome instability mediated by the Top1 mutant, we constructed two other yeast strains with *TOP1* mutant alleles (**Table 1**). yTop1Y740Stop is analogous to the human Top1W763Stop mutant identified in a lung cancer patient treated with the CPT-derivative irinotecan and is predicted to have reduced catalytic activity, while yTop1S733E is a duplex DNA binding mutant analogous to the human Top1T729E mutant that confers CPT-resistance when expressed in yeast [32, 33]. Construction of both the *yTOP1Y740STOP* and *yTOP1S733E* strains allowed us to distinguish between DNA cleavage-defect and DNA binding-defect as the cause of G4-associated genomic instability. Expression of all C-terminally 3XFLAG-tagged Top1 mutants enabled their detection by western blotting (Figure S1A-B). yTop1Y740Stop yielded a significantly lower steady-state protein level than wild type (WT) yTop1, yTop1Y727F, or yTop1S733E. In yeast cells where the repair of Top1cc is partly disabled by deletion of *MUS81*, expression of yTop1Y740Stop or yTop1S733E led to CPT-resistance as expected from the study of analogous human mutations (Figure S1C).

**TABLE 1.** **Top1 mutants studied.** Catalytic Top1 mutants (Y723F/Y727F and W736Stop/Y740Stop) can bind but not cleave duplex DNA while DNA binding mutants (T729E/S733E) cannot bind duplex DNA.

**Human Mutant**	**Yeast Mutant**	**DNA Binding**	**DNA Cleavage**	**CPT Resistance**	**Source**
Y723F	Y727F	Yes	No	Yes	Top1 crystal structure study [44]
W736STOP	Y740STOP	Yes	No	Yes	Non-small cell lung cancer patient treated with irinotecan [32]
T729E	S733E	No	No	Yes	Study characterizing CPT-resistance conferring resistance [33]

To further confirm that the newly constructed yeast Top1 mutants are functionally defective, we used a yeast genetic assay where four AG repeats were inserted into the reversion window of the *lys2*Δ*A746,NR* frameshift allele [34, 35]. As reported earlier, the reversion mutation at *lys2*Δ*A746,NR,(AG)*_*4*_ allele, which requires a net of two base pair deletion, is dependent upon the presence of functional Top1, particularly when RNase H2 is absent. RNase H2 complex normally keeps genomic DNA free of ribonucleotides by initiating the excision repair of ribonucleotides incorporated during replication. In *rnh201*Δ backgrounds, ribonucleotides remaining embedded in DNA are subsequently cleaved by Top1 and, in case of repetitive sequences, frequently lead to slippage or frameshift mutations. For the *lys2*Δ*A746,NR,(AG)*_*4*_ allele, processing of the Top1-nicked DNA ends leads to two base pair deletions within the (AG)_4_ repeats [34, 35]. As reported earlier, absence of functional Top1, as in *top1*Δ backgrounds, results in a >200-fold decrease in the mutation rate at the *lys2*Δ*A746,NR,(AG)*_*4*_ allele [35] (Table S2). Since mutagenic processing of ribonucleoside monophosphates (rNMPs) is dependent on Top1's ability to cleave DNA, we expressed Top1 mutants in the *lys2*Δ*A746,NR,(AG)*_*4*_ *rnh201*Δ background to measure Top1-dependent rNMP cleavage as a proxy for Top1 catalytic activity. *LYS2* reversion mutation rates of the *yTOP1Y727F, yTOP1Y740Stop,* and *yTOP1S733E lys2*Δ*A746,NR,(AG)*_*4*_ *rnh201*Δ were all statistically indistinguishable from the *top1*Δ *lys2*Δ*A746,NR,(AG)*_*4*_ *rnh201*Δ rate, confirming all three of the yeast Top1 mutants are catalytically inactive (Table S2).

**Figure 1:**
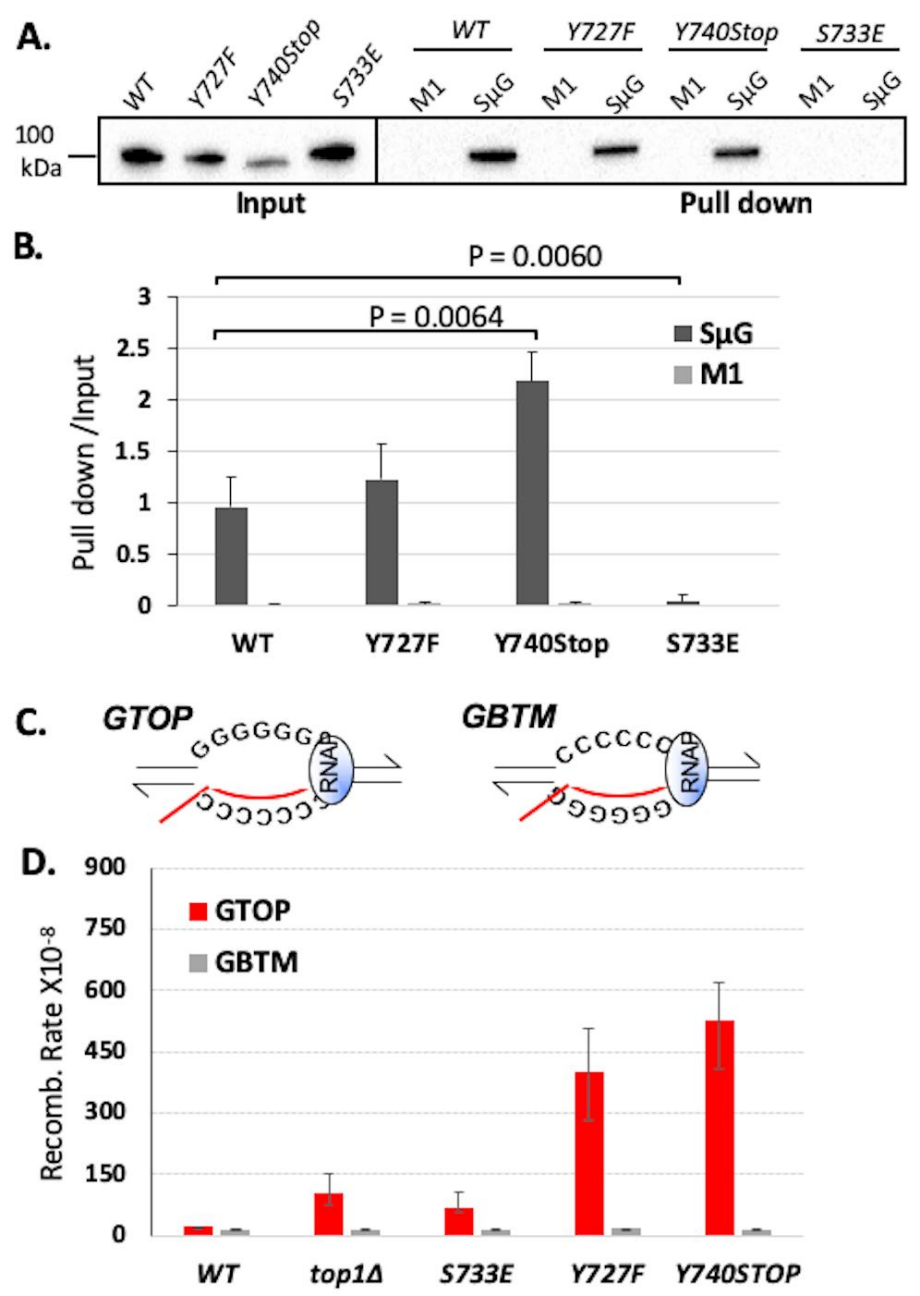
FIGURE 1: Top1 mutants bind G4 oligos *in vitro* and increase G4-induced recombination in yeast. **(A)** Western blot from biotinylated oligo pulldown assay performed with streptavidin magnetic beads and yeast whole cell lysates expressing FLAG-tagged Top1 proteins. Blot was probed with an α-FLAG-HRP (Sigma) antibody. Quantification of binding is listed below blot and was calculated by dividing input pixel intensities from pull down pixel intensities. **(B)** Quantification (means and standard deviations of pull-down pixel intensities normalized to input pixel intensities) of western blots from three *in vitro* biotin oligo pulldown assays with SμG and M1 oligos and Top1 proteins performed as in A. Vertical error bars only show positive standard deviations. P-value derived from students T-test (GraphPad Prism). **(C)** Transcription orientations and the guanine-runs in the Sμ-containing recombination reporter constructs. Guanine-runs are located on the non-transcribed or the transcribed strand in the *pTET-lys2-GTOP* or *pTET-lys2-GBTM* construct, respectively. The red line within the transcription bubble indicates the transcript. RNA polymerase complex is indicated as blue oval in front of the transcription bubble. **(D)** Recombination rates of Top1 mutant-expressing yeast strains. Rates are considered statistically significantly different if their 95% confidence intervals (shown as error bars) do not overlap [74].

**Figure 2:**
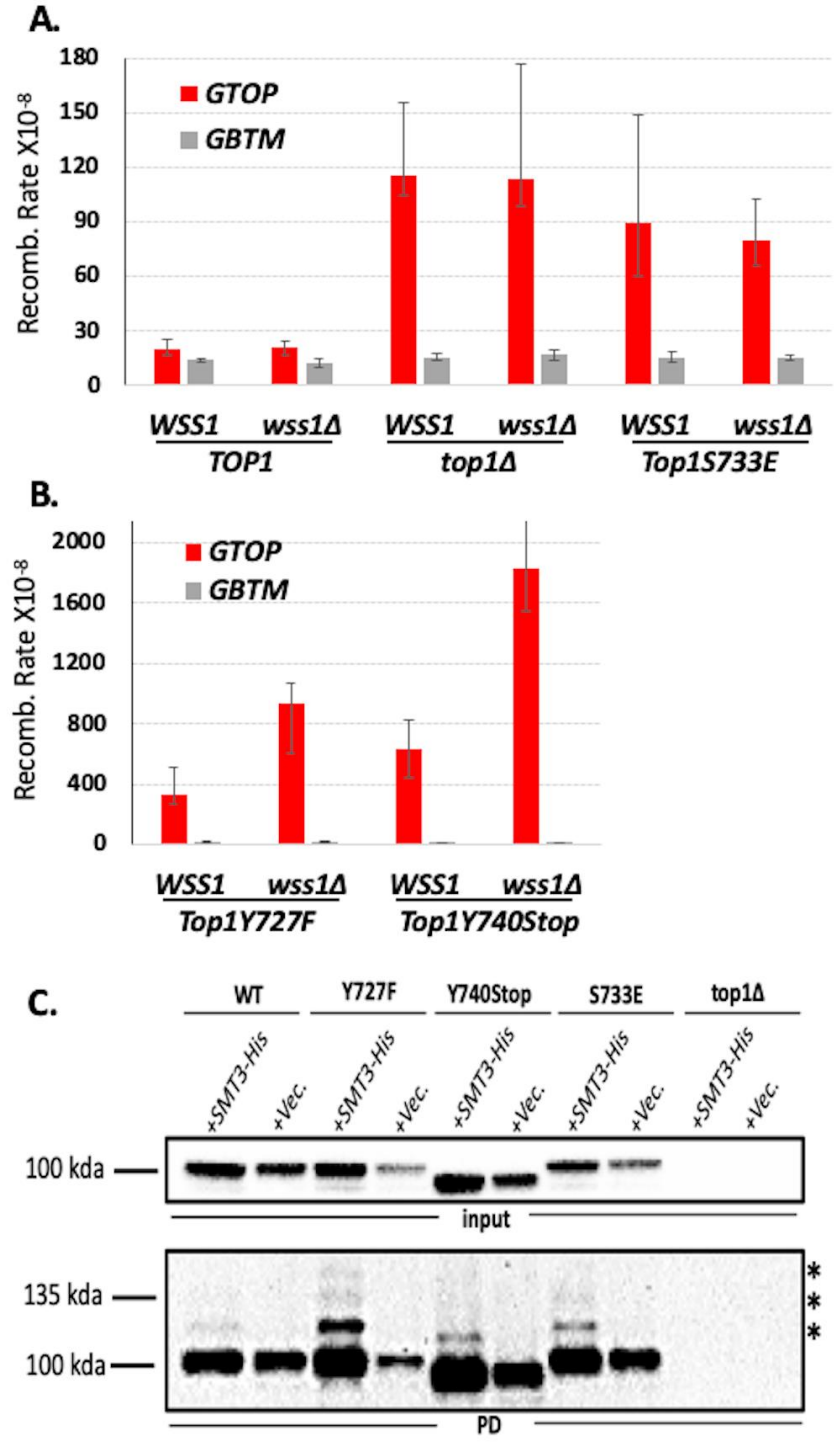
FIGURE 2: Deletion of *WSS1* increases G4-induced recombination in Top1 catalytic mutant strains. **(A, B)** Recombination rates of *WSS1* and *wss1*Δ yeast strains expressing indicated Top1 alleles. Rates are considered statistically significantly different if their 95% confidence intervals (shown as error bars) do not overlap [74]. **(C)** Top1-SUMO pull down experiment. All experiments were carried out in *wss1*Δ strains expressing C-terminally 3X-FLAG-tagged Top1 proteins and N-terminally 7XHis-tagged Smt3. SUMO-modified proteins were pulled down using Ni+ beads and Top1 proteins detected by western blotting with α-FLAG-HRP antibody. Top panel – inputs; bottom panel – pull down samples (PD). * denoted bands are mono- or poly-SUMOylated Top1 proteins.

We next measured the ability of the Top1 mutants to bind to a duplex DNA oligo using an *in vitro* biotinylated oligonucleotide (oligo) pulldown assay. The biotinylated SS oligo was annealed to the non-biotinylated RC-SS oligo to create a duplex substrate that could be used to pull down the Top1 mutants from yeast whole cell lysates if binding occurs (Figure 2SA). yTop1Y727F and yTop1Y740Stop cleavage-defective mutants bound duplex DNA while the yTop1S733E DNA binding-defective mutant did not (Figure 2SB). When we performed *in vitro* DNA binding assays using an oligo that is G4-capable (SμG; **Table 2**), WT yTop1, yTop1Y727F, and yTop1Y740Stop all formed stable complexes with the SμG oligo but not to M1 oligo where two guanine-runs present in SμG were interrupted (**Figure 1A** and **1B**). However, yTop1S733E lacked appreciable binding to SμG oligo, indicating that Top1 duplex DNA binding mutants do not form stable complexes with G4 structures like WT yTop1 or the Top1 cleavage-defective mutants yTop1Y727F and yTop1Y740Stop.

**TABLE 2.** **Oligonucleotides used in binding assays.** SμG oligo can adopt a G4 conformation while the M1 oligos cannot. Guanine-runs in SμG oligo are in bold. G>A mutations in M1 oligo introduced to disrupt guanine-runs are indicated as lowercase letters.

**Oligo**	**Sequence**	**Length**	G-score*
SμG	5′GAGCT**GGGG**TGAGCT**GGG**CTGAGCT**GGGG**TGAGCT**GGG**CTGAGCT	45 nt	70
M1	5′GAGCTGaGGTGAGCTGGGCTGAGCTGaGGTGAGCTGGGCTGAGCT	45 nt	N/A

* G-score was calculated using QGRS Mapper (https://bioinformatics.ramapo.edu/QGRS/index.php; parameters: Max Length – 44, Min G-Group Size – 3, and Loop Size – 0 to 10).

### Top1 cleavage-defective mutants increase G4-induced recombination more than a Top1 duplex DNA binding mutant

We examined the effect of transcription on G4-induced recombination using a reporter construct containing a model G4-motif from the mouse immunoglobulin switch region Mu (*SμG4*). In this reporter, a segment of *SμG4* was integrated into the yeast genome within the *LYS2* gene under the control of tetracycline/doxycycline-repressible promoter (*pTET-lys2*) [23]. The *SμG4*-motif was inserted into the *pTET-lys2* allele in two different transcriptional orientations, each disrupting the *LYS2* open reading frame (ORF). In *GTOP* orientation (*pTET-lys2-GTOP*), the guanine runs of *SμG4* are on the non-transcribed strand (NTS) which is transiently single-stranded during transcription, facilitating G4-formation (**Figure 1C**). In *GBTM* orientation (*pTET-lys2-GBTM*), the guanines of *SμG4* are on the transcribed strand (TS), which is based paired with the nascent mRNA during transcription and thus not likely to adopt G4-conformations. In this reporter assay, DNA strand breaks at *SμG4 GTOP* or *GTBM* are repaired via recombination utilizing a truncated genomic copy of *LYS2*. The recombination rate at *SμG4* can thus be inferred from the emergence of Lys+ recombinants. Any factor that affects G4-formation or -stability involves recombination starting at *GTOP*, but not *GBTM* [23]. We have previously shown that more recombination occurs at *pTET-lys2-GTOP* than at -*GBTM* under active transcription, and that this difference in recombination is increased significantly by the absence of Top1, supporting the hypothesis that Top1 functions to prevent co-transcriptional G4-formation by averting excessive torsional stress on DNA [28].

Using the *pTET-lys2-GTOP* and -*GBTM* reporter strains, we first confirmed that the C-terminal 3X-FLAG tag does not affect the functioning of WT yTop1 in a recombination reporter assay (Figure S3). For yeast strains expressing yTop1Y727F or yTop1Y740Stop mutants, the recombination rates for the *pTET-lys2-GTOP* reporter construct were ~3.8- or ~4.9-fold higher than the rate for the *top1*Δ strain, respectively (**Figure 1D**). The *pTET-lys2-GTOP* recombination rate in the yTop1S733E-expressing strain was significantly lower than for the yTop1Y727F- or yTop1Y740Stop-expressing mutants and was statistically similar to the *top1*Δ strain. These results indicate that the exacerbated genomic instability at G4s in yeast cells expressing the Top1 cleavage-defective mutants yTop1Y727F or yTop1Y740Stop is due to a mechanism distinctly different from the *top1*Δ or the DNA binding-defective mutant yTop1S733E-expressing cells. Importantly, the effect of Top1 mutation on recombination is G4-specific since the recombination rates at the *pTET-lys2 GBTM* reporter construct did not significantly change by the expression of any of the Top1 mutants (**Figure 1D**). When transcription through *SμG4* was suppressed by adding doxycycline to culture media, the *pTET-lys2-GTOP* recombination rates of the *top1*Δ*, yTOP1Y727F, yTOP1Y740Stop,* and *yTOP1S733E* strains were slightly elevated relative to the WT *GTOP* rate but to much less extant than without doxycycline (Figure S4). Thus, the effect of *TOP1*-deletion and mutation on *GTOP* recombination is largely transcription-dependent.

### The DNA-dependent protease Wss1 alleviates G4-associated genome instability exacerbated by cleavage-defective Top1 mutants

*WSS1* encodes a DNA-dependent protease that degrades proteins bound to DNA [36, 37]. Top1ccs trapped on DNA are Wss1's best characterized substrate. Deletion of *WSS1* did not affect the recombination in WT or *top1*Δ backgrounds (**Figure 2A**) but significantly elevated the rates of recombination at the *pTET-lys2-GTOP* construct only in y*TOP1Y727F- or* y*TOP1Y740Stop-*expressing strains (**Figure 2B**). These results support that the cleavage-defective Top1 mutants trapped on *SμG4 in vivo* were substrates for Wss1. *WSS1-*deletion did not affect the rate of recombination at the *pTET-lys2-GTOP* construct in *yTOP1S733E-*expressing strains, consistent with the *in vitro* oligo binding assays showing that yTop1S733E does not bind G4s formed on *SμG4 GTOP* (**Figure 2A**). Additionally, *WSS1*-deletion did not affect the recombination rate at the *pTET-lys2-GBTM* construct in any of the strains (**Figure 2A** and **2B**), indicating that Wss1 has a specificity for proteins bound to G4s in our fluctuation assays and that the Top1 catalytic mutants are not persistently bound to *SμG4* when G4-formation is not supported. In *wss1*Δ strains, Top1 WT and mutant proteins are SUMOylated with Top1Y727F being most extensively modified by SUMO ligation (**Figure 2C**).

### N-terminal domain and RGG repeats of Nsr1 are required for synergistic elevation of G4-induced genomic instability in cells with a Top1 cleavage-defective mutant

**Figure 3:**
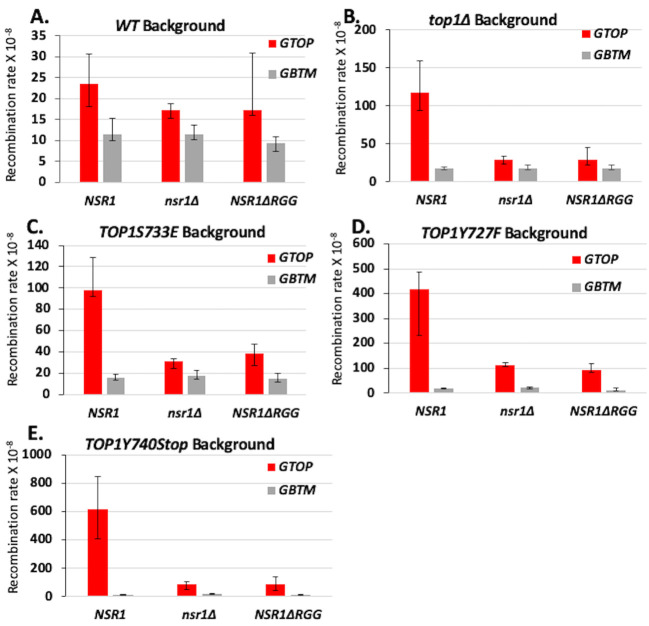
FIGURE 3. Deletion of *NSR1* and deletion of the RGG domain of Nsr1 reduces G4-induced recombination in Top1 mutant strains. *A.-E.* Recombination rates of *NSR1, nsr1Δ,* and *nsr1ΔRGG* strains expressing indicated Top1 alleles. Rates are considered statistically significantly different if their 95% confidence intervals (shown as error bars) do not overlap [74].

The yeast protein Nsr1, a homolog of human nucleolin (NCL), is a G4-binding protein required for G4-induced recombination in *top1*Δ cells [19]. Nsr1 binds to co-transcriptionally formed G4s in the absence of yTop1 leading to replication stalling along the *SμG4-*containing *pTET-lys2-GTOP* locus. We deleted *NSR1* in the Top1 mutant backgrounds and measured recombination at the *SμG4-*containing recombination reporters. *NSR1*-deletion significantly reduced recombination rates at the *pTET-lys2-GTOP* reporter in all backgrounds (**Figure 3A-E**). Deletion of *NSR1* in *top1*Δ and *yTOP1S733E* strains reduced recombination at the *pTET-lys2 GTOP* reporter to WT background levels (**Figure 3B** and **3C**). In cells expressing *yTOP1Y727F* or *yTOP1Y740Stop,* recombination rates at the *pTET-lys2 GTOP* reporter were reduced by *NSR1*-deletion but still significantly higher than WT and similar to those measured in *top1*Δ (**Figure 3A-B** and **Figure 3D-E**). *NSR1-*deletion did not affect the recombination rate at the *pTET-lys2-GTOP* reporter in a WT background or the recombination rates at the *pTET-lys2-GBTM* reporter in any background tested (**Figure 3A-E**).

The C-terminally located RGG repeats of Nsr1 are required for high-affinity G4-binding [19]. When we expressed a truncated form of Nsr1 lacking the RGG domain (Nsr1ΔRGG = Nsr1 amino acid residues 1-350), recombination at the *pTET-lys2-GTOP* reporter was significantly reduced in all Top1 mutant strains (**Figure 3C-E**). As observed in the above experiments with *NSR1*-deletion, recombination at the *pTET-lys2 GTOP* reporter was reduced to WT levels in the *yTOP1S733E NSR1*Δ*RGG* strain but remained above WT levels in *yTOP1Y727F NSR1*Δ*RGG* and *yTOP1Y740Stop NSR1ΔRGG* strains (**Figure 3A** and **Figure 3C-E**). For the *pTET-lys2-GBTM* reporter construct, recombination rates were not affected by either *NSR1-*deletion or deletion of Nsr1's RGG domain in any background, except in the y*TOP1Y740Stop nsr1*Δ strain where the recombination rate was significantly higher than the rate in the y*TOP1Y740Stop* background (**Figure 3A-E**).

**Figure 4:**
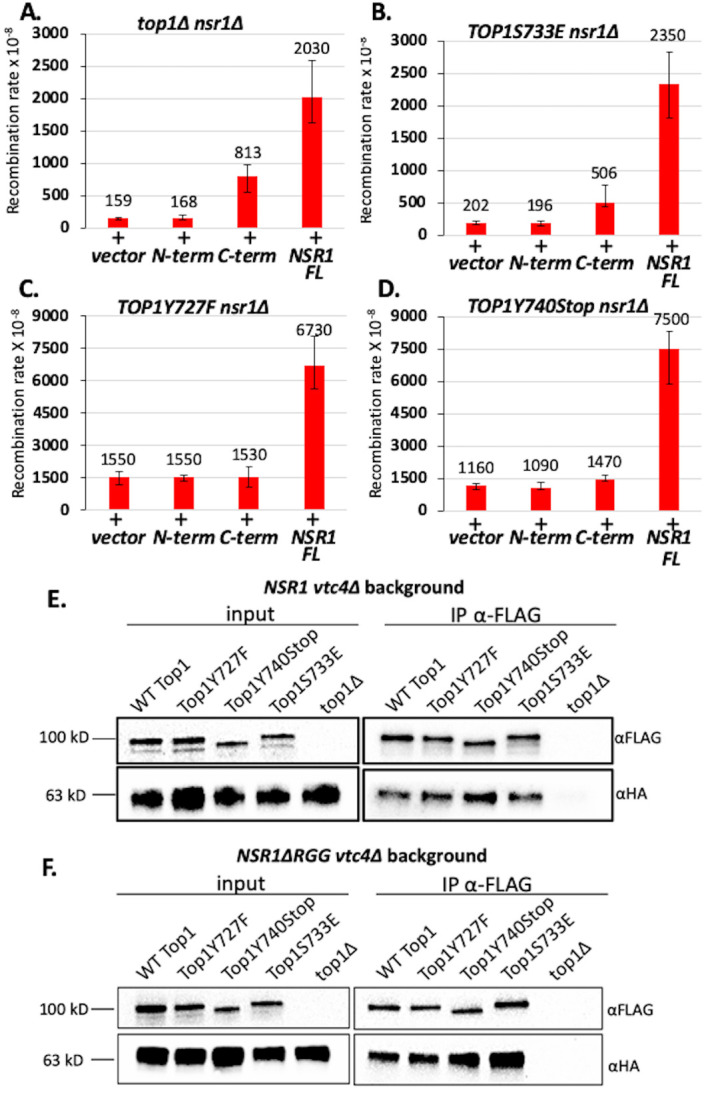
FIGURE 4: Expression of full-length *NSR1* is required to greatly exacerbate G4-induced recombination in *TOP1Y727F nsr1*Δ and *TOP1Y740Stop nsr1*Δ strains. (A-D) Recombination rates of *top1*Δ *nsr1*Δ (A), *TOP1S733E nsr1*Δ (B), *TOP1Y727F nsr1*Δ (C), or *TOP1Y740STOP nsr1*Δ (D) yeast strains expressing the indicated Nsr1 constructs. Rates are listed above their respective bars and are considered statistically significantly different if their 95% confidence intervals (shown as error bars) do not overlap. N-term = pADH1-Nterm Nsr1, C-term = pADH1-Cterm Nsr1, NSR1 FL = pADH1-Nsr1, and vector = pRS426. **(E, F)** Co-immunoprecipitation (co-IP) experiments conducted with *vtc4*Δ yeast strains expressing 3XFLAG-tagged Top1 proteins and either 6X-HA-tagged full-length Nsr1 (E) or NsrΔRGG (F). Pull down was carried out with αFLAG antibody-coated agarose beads. Blots were probed with either αFLAG or αHA antibodies. Quantification of binding was calculated by dividing FLAG IP pixel intensities from HA-IP pixel intensities and presented in graphs in Figure S10.

We additionally performed functional complementation experiments in *nsr1*Δ strains. The N-terminus of Nsr1 (N-term Nsr1; amino acids 1-171), the C-terminus of Nsr1 (C-term Nsr1; amino acids 171-414), or full-length Nsr1 (Nsr1 FL; amino acids 1-414) was expressed in *nsr1*Δ cells expressing one of the Top1 mutants using a high copy *2μ* plasmid and verified by western blot analyses (Figure S5). Expression of C-term Nsr1 in a *top1*Δ *nsr1*Δ background increased the recombination rate at the *pTET-lys2-GTOP* compared to either the vector control or the N-term Nsr1 (**Figure 4A**) [19]. Similar effects were observed in the *yTOP1S733E nsr1*Δ strain, where expression of Nsr1 C-term, but not Nsr1 N-term, increased recombination rates at the *pTET-lys2-GTOP* (**Figure 4B**). Notably, expression of full-length Nsr1 resulted in greater elevation in recombination rates at the *pTET-lys2-GTOP* than C-term Nsr1 in both *top1*Δ *nsr1*Δ and *yTOP1S733E nsr1*Δ backgrounds. In the *yTOP1Y727F nsr1*Δ strain, the expression of neither C-term Nsr1 nor N-Term Nsr1 significantly changed recombination rates at the *pTET-lys2-GTOP* (**Figure 4C**). Only expression of full-length Nsr1 (Nsr1 FL) elevated the recombination rate at the *pTET-lys2-GTOP* above the control in the *yTOP1Y727F nsr1*Δ strain. Similar results were seen with the *yTOP1Y740Stop nsr1*Δ strain, where expression of Nsr1 FL resulted in a ~6.5-fold elevation in recombination rates at the *pTET-lys2-GTOP* relative to the vector control (**Figure 4D**). In the *yTOP1Y740Stop nsr1*Δ *GTOP* strain, expression of C-term Nsr1 led to a significant but relatively moderate increase in the recombination rate at the *pTET-lys2-GTOP* relative to the vector control - ~1.3-fold increase compared to the 5- or 2.5-fold increase seen with the expression of C-Term Nsr1 in *top1*Δ *nsr1*Δ or *yTOP1S733E nsr1*Δ backgrounds, respectively (**Figure 4A, B**, and **D**). In summary, the Top1-interacting N-terminus of Nsr1 is required to exacerbate G4-associated genome instability induced by yTop1Y727F or yTop1Y740STOP.

### Top1 mutants interact with Nsr1

Because the complete deletion of *NSR1* or the expression of the Nsr1ΔRGG-truncated protein significantly reduces G4-associated recombination in cells expressing Top1 cleavage-defective mutants, we tested whether Top1 cleavage-defective mutants can bind Nsr1 to cooperatively interact on G4 DNAs. Indeed, a G4 DNA/Nsr1/Top1-mutant complex could be a potent block to DNA replication and thus explain the exacerbated G4-induced instability observed in the presence of Top1 catalytic mutants and Nsr1. While interactions between WT Top1 and Nsr1 have been shown [38, 39], interactions between a Top1 mutant and Nsr1 have not been previously reported.

For co-immunoprecipitation (co-IP) experiments, C-terminally 3XFLAG-tagged Top1 mutants and C-terminally 6XHA-tagged Nsr1 were expressed in yeast cells. First, we showed that Nsr1-6XHA is stably expressed in all Top1 mutant backgrounds (Figure S6B) and that the C-terminal 6XHA-tag on Nsr1 does not affect G4-induced recombination at the *pTET-lys-GTOP* reporter (Figure S7). All co-IP experiments were conducted in a *VTC4*-deletion background because Top1/Nsr1 interactions are not easily detectable in the presence of Vtc4 protein, which is required for the synthesis of post-translational poly-phosphorylation (Figure S8) [39, 40]. *VTC4-*deletion did not affect the rate of recombination at *pTET-lys2-GTOP* reporter in any strain expressing either full-length Nsr1 or the Nsr1ΔRGG truncation (Figure S9). In *vtc4*Δ backgrounds, Top1/Nsr1 interactions were detected in lysates prepared from all Top1 protein strains tested (**Figure 4E**) and quantification of multiple co-IP experiments revealed that all the Top1 proteins tested undergo a similar level of interaction with Nsr1 (Figure S10A). Interaction between Nsr1ΔRGG and Top1 mutants were tested through co-IP experiments in *vtc4*Δ strains (**Figure 4F**); all three Top1 mutants, i.e. Top1Y727F, Top1Y740STOP, and Top1S733E, interacted with Nsr1ΔRGG to a similar degree (Figure S10B). Our composite co-IP data demonstrate that neither Top1 mutation nor deletion of Nsr1's RGG domain impacts the Top1-Nsr1 interaction.

### Somatic Top1 mutations are associated with high mutation rates in cancer

**Figure 5:**
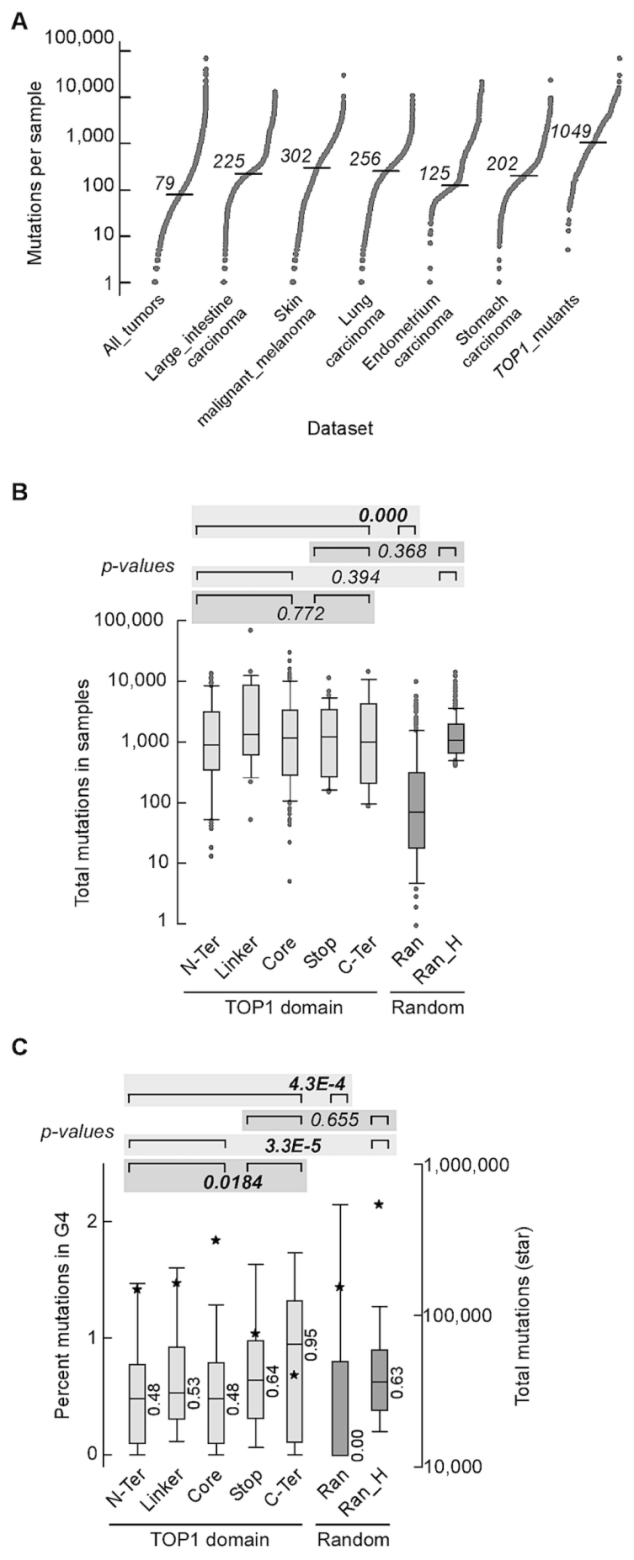
FIGURE 5: Somatic mutations in the *TOP1* gene are associated with high mutation rates in cancer. **(A)** S-plots of number of mutations exome-wide. *All_tumors*, 35,887 samples from Cosmic v.94 comprising all exome-wide screens (i.e. field “Genome-wide screen” corresponding to “y”) and non-redundant sample codes; *Large_intestine carcinoma*, 2,355 samples from All_tumors comprising carcinomas of the large intestine, 44 of which had mutations in *TOP1*; *skin_malignant_melanoma*, 1,372 samples from All_tumors with malignant melanoma of the skin, 31 with mutations in *TOP1*; *Lung_carcinoma*, 2,512 lung carcinoma samples from All_tumors, 27 with TOP1 mutations; *Endometrium_carcinoma*, 606 samples from All_tumors with carcinoma in the lining of the womb, 23 with mutations in *TOP1*; *Stomach_carcinoma*, 1,349 samples from All_tumors with stomach carcinoma, 16 with *TOP1* mutations; *TOP1_mutants*, all 239 samples from All_tumors with mutations in *TOP1*. *Horizontal dash*, median. **(B)** Box plot shows number of mutations in samples carrying mutations in different *TOP1* domains. *N-Ter*, 61 samples with mutations in the amino terminus domain (median = 878); *Linker*, 26 samples with mutations in the Linker (median = 1321.5); *Core*, 102 samples with mutations in the Core domain (median = 1,149); *Stop*, 36 samples with nonsense mutations (median = 1,219); C-Ter, 14 samples with mutations in the carboxyl terminus domain (median = 982.5); *Ran*, 300 samples chosen at random among All_tumors (median = 73.5); *Ran_H*, random_high: a pool of 1,500 random samples with at least 400 mutations each were chosen from All_tumors and 300 entries were then chosen from the pool, after removing samples with identical codes but assigned to different types of tumor in COSMIC (median = 1049). P-values were from Wilcoxon tests. For the purpose of single Wilcoxon tests, we combined the numbers of mutations and numbers of samples when applicable. All p-values are shown in Table S14. **(C)** Box plots of mutations at G4 tracts. For each sample the value refers to the percent mutations that overlapped with G4-forming repeats. Data sets are as in panel B. *Stars*, total number of mutations; median values are shown. P-values from Wilcoxon tests. For the purpose of single Wilcoxon tests, we combined the percent mutations at G4 and numbers of samples when applicable. All p-values are shown in Table S15. Outliers were removed for clarity.

Next, we assessed the effect of *TOP1* somatic mutations on mutation rates and mutations at G4 potential non-B DNA-forming sequences (PONDS) in a mammalian system by analyzing 15,196,274 exome-wide mutations, mostly single-base substitutions and small indels, in 35,887 tumor samples representing 37 different tissues (All_tumors – Table S3). DNA strand breaks or replication forks stalled at G4 are expected to be processed by one of several repair pathways, including highly error-prone processes such as non-homologous end joining (NHEJ), microhomology-dependent alternative end-joining (alt-EJ), or break-induced replication (BIR), leading to elevation of small indels and base substitutions [41–43]. Mutations in *TOP1* were partitioned into five groups, representing four different protein domains, i.e. amino terminus (N-Ter), linker, core, carboxy terminus (C-Ter), and truncation (Stop) mutations (Table S4). The number of samples with *TOP1* mutations ranged from 14 for the C-Ter domain to 106 for the Core domain. The median number of mutations for the All_tumors dataset was 79 (**Figure 5A**), ranging from 1 to 69,039 (Table S3). Interestingly, the set of 239 samples with *TOP1* mutations (Top1_mutants) exhibited a median number of mutations ~12-fold higher than All_tumors (**Figure 5A**). To rule out the tumor types as a variable, we narrowed the analysis to five types of tumor with the greatest number of *TOP1* mutations – large intestine carcinoma, skin malignant melanoma, lung carcinoma, and stomach carcinoma (Table S5). The median numbers of mutations in these five types of tumors, ranging from 125 in endometrium carcinoma to 302 in skin malignant melanoma, were significantly higher than that in All_tumors but still ~8 to 3-fold lower than in the tumors with Top1 mutations (**Figure 5A**). For *TOP1*, mutations in the carboxy terminal domain (C-Ter) as well as those leading to truncated protein (Stop) are most likely to affect its catalytic function. We thus combined N-Ter, Linker and Core into one group and C-Ter and Stop into another. The number of mutations for the combined N-Ter, Linker and Core and for the combined C-Ter and Stop was also higher than the 8,194 samples comprising the five types of tumor with the greatest number of *TOP1* mutations (p-values of 0.000 and 8.4E-11, respectively, Table S6).

To statistically assess if the number of mutations in *TOP1* mutant samples was higher than expected, we generated a set of 300 samples taken randomly from All_tumors (Ran). Also, from All_tumors, a second set of 300 samples with a median number of mutations comparable to that of the TOP1_mutants were selected (Ran_H). The number of mutations for the combined 239 TOP1_mutants was significantly higher than for the Ran set (**Figure 5B**). In contrast, no differences were noted when we compared the number of total mutations in N-Ter, Linker and Core sample sets together with C-Ter and Stop together, or between each of these two subsets and Ran_H (**Figure 5B**). To evaluate if the hypermutation associated with TOP1-mutant tumors impacted G4 PONDS, we computed the percent base-changes, seen in tumors relative to the reference genome, that occurred at G4 PONDS relative to genome-wide (Table S7 – S13). For the combined 239 TOP1_mutants, the percentages of mutations at G4 PONDS were higher than for the random sample (Ran; medians 0.52 and 0.00, respectively, **Figure 5C**). Interestingly, percentages of mutation at G4 PONDs were significantly higher for the combined C-Ter and Stop than for the combined N-Ter, Linker and Core (medians 0.67 and 0.48, respectively). For the Ran_H control set, percentages of mutation at G4 PONDs were higher than the combined N-Ter, Linker, and Core, but were indistinguishable from the combined C-Ter and Stop. From these data, we conclude that tumors with mutations in *TOP1* carry an exceeding high mutations burden in cancer; this high mutation burden appears to be linked directly to the mutated TOP1 since mutations that retain an intact carboxy terminal domain exerts a less profound effect on base changes at G4 PONDS than those that compromise it.

## DISCUSSION

Co-transcriptional helical stress, promoting formation of DNA secondary structures including G4 DNA, is expected to accumulate when Top1's normal function of relieving DNA supercoils by binding and cleaving DNA is completely disrupted [44]. Multiple studies including our previous works support the notion that Top1 functions to prevent co-transcriptional G4-formation by removing negative helical stress [27, 28]. It remained to determine how the expression of catalytic or DNA binding Top1 mutants found in CPT-resistant cancer cells would affect DNA aberrations at G4s. The C-terminal domain of Top1 partly forms a tight loop around duplex DNA and contains catalytically important residues, including the phosphotyrosyl bond-forming tyrosine (Y727 and Y723 in yeast and human, respectively) [44]. The high conservation between yeast and human C-terminal domains of Top1 allowed us to measure the effect of C-terminal Top1 mutants found in CPT-resistant human cancer cells on G4-induced instability by expressing the analogous mutants in yeast.

In an earlier study, we found that the expression of catalytically null mutant Top1Y727F results in severely elevated genome instability at G4 DNA-forming recombination reporter in yeast cells. Unexpectedly, the rate of G4-associated recombination in Top1Y727F-expressing cells was significantly higher than in *top1*Δ cells [28]. Here, we expressed another cleavage defective mutant, yTop1Y740Stop, and observed similarly acute elevation of G4-associated instability, whereas the DNA binding-defective mutant yTop1S733E had a more moderate effect on G4-induced instability (**Figure 1D**). Specifically, yTop1Y727F and yTop1Y740STOP expression resulted in *pTET-lys2-GTOP* recombination rates that were around 5.9-fold and 7.8-fold higher than yTop1S733E expression. For the *pTET-lys2-GBTM* reporter where G4 DNA formation was unfavorable, Top1 mutants regardless of the specific mutations, did not impact the rate of recombination, irrespective of the specific mutations, suggesting that the effect of Top1 mutations is G4-specific.

Multiple studies have documented interactions of Top1 with G4s [29–31, 45]. Therefore, we postulated that the severe elevation in recombination rates at *pTET-lys2-GTOP* reporter observed upon expression of Top1 cleavage-defective mutants Top1Y727F and Top1Y740STOP was the result of the binding and stabilization of co-transcriptionally formed G4s. It is possible that, while WT Top1 undergoes transient interactions with G4s *in vivo,* the inability to cleave DNA subsequent to binding could leave Top1Y727F and Top1Y740STOP trapped on G4s that form during transcription, significantly disrupting replication. Cleavage-defective mutants yTop1Y727F and yTop1Y740STOP, but not yTop1S733E, bind a G4-forming oligo but not the control M1 oligo *in vitro* (**Figure 1A** and **1B**). This result is in an agreement with previous published data showing purified calf thymus Top1 has a specificity for G4-capable oligos over non-G4 capable DNA substrates [46]. Our *in vitro* binding data are in line with the *GTOP* recombination data, where Top1 catalytic mutants induce significantly greater recombination at *SμG4* than the Top1 duplex DNA binding mutant (**Figure 1D**). Furthermore, when combined with the low steady-state protein level of yTop1Y740Stop (Figure S1A-B), the high G4-induced recombination in yeast cells expressing this Top1 mutant (**Figure 1D**) supports a possible dominant negative phenotype of hTop1W736Stop.

*WSS1* encodes a SUMO-dependent metalloprotease [37, 47]. Wss1 and its mammalian homolog SPRTN degrade proteins forming covalent, irreversible complexes with DNA or DNA–protein cross-links (DPCs) and thus suppress genome instability incurred by DPC-induced replication impediment [48]. Besides DPCs occurring as trapped enzymatic intermediates, they can be generated by crosslinking of DNA to protein by reactive agents such as formaldehyde and metal ions. Most Wss1 targets are SUMOylated and both Wss1 and SPRTN recognize and target Top1 cleavage complexes (Top1ccs) that are post-translationally modified [36, 37, 49, 50]. Top1ccs have been shown to be SUMOylated [51], and human (Top1Y723F) and yeast (Top1Y727F) Top1 catalytic mutants were found to be more heavily SUMOylated than WT Top1 proteins [52–54]. Even through non-covalent interaction, certain proteins such as yeast Fob1 or *E. coli* Tus can form effective DPC-like stable, high-affinity complexes with DNA that block replication fork movement [55]. Recently, histones in such non-covalent DPCs were shown to be substrates for Wss1 [56]. We postulate that Top1 mutants Y727F and Y740STOP, if forming replication barriers through high-affinity interaction with G4 DNA, could be SUMOylated and targeted by Wss1-dependent proteolysis.

Upon deletion of *WSS1*, the recombination rates at *pTET-lys2-GTOP* in yTop1Y727F- and yTop1Y740STOP-expressing strains were each elevated by ~3-fold (**Figure 2B**). Since the *pTET-lys2-GTOP* recombination rates in WT and *top1*Δ were not significantly changed by *WSS1*-deletion (**Figure 2A**), the effect of *WSS1*-deletion on G4-induced recombination is specific to the Top1 cleavage-defective mutant. Consistent with the data where yTop1S733E does not appear to be tightly bound to G4 DNA *in vitro* and where the recombination at *pTET-lys2-GTOP* did not further elevate upon expression of yTop1S733E in *top1*Δ background, WSS1-deletion did not affect the recombination rate in the yTop1S733E-expressing strain (**Figure 2A**). These results provide indirect evidence that Top1 mutants Y727F and Y740STOP form stable complexes with G4 DNA *in vivo* and further indicate that Wss1 can partly suppress the genome instability instigated by Top1 cleavage-defective mutants in complex with G4 DNA.

Nsr1 is the yeast homolog of human nucleolin, a clinically relevant protein that exhibits altered expression and localization in cancer cells [57–59]. While the primary functions of nucleolin and Nsr1 are in pre-ribosomal RNA processing [60], both nucleolin and Nsr1 are G4-binding proteins [19, 61, 62]. The biological consequences of nucleolin-G4 DNA interaction was demonstrated by transcriptional change in several oncogenes including *MYC* upon binding of nucleolin to G4s present in the promoter [63, 64]. Our group recently published the first results showing that, in *top1*Δ cells, Nsr1-G4-binding is responsible for the elevated recombination at the *SμG4-*containing *pTET-lys2-GTOP* reporter as well as for the significant lag in DNA replication timing observed at the *SμG4-*containing genomic locus [19]. In the current study, we show that *NSR1*-deletion significantly reduces the G4-associated genomic instability observed in all Top1 mutant yeast strains tested (**Figure 3C-E**). In yTop1S733E-expressing cells, the decrease in recombination rates due to deletion of *NSR1* resembles that seen in the *top1*Δ background, indicating that the DNA-binding defect in this Top1 mutant essentially mimics the lack of functional Top1 with no additional detrimental effect. However, in yTop1Y727F- and yTop1Y740Stop-expressing cells, rates of recombination at the *pTET-lys2-GTOP* reporter were reduced upon deletion of *NSR1* but still significantly higher than in WT background. This indicates that Top1 cleavage-defective mutants can instigate G4-associated instability even in the absence of Nsr1.

The C-terminal RGG domain of human nucleolin is important for the protein's high-affinity interaction with G4 structures [61, 65]. Moreover, phenylalanine residues in the RGG domain of nucleolin participate in G4-binding and G4-folding, as shown through electrophoretic mobility-shift assays and circular dichroism spectroscopy experiments [66]. Our own work uncovered that the C-terminally located RGG-domain of Nsr1 is required for G4-binding and the induction of co-transcriptional G4-induced instability in the absence of Top1 [19]. In the current study, we show that deletion of the RGG domain of Nsr1 also significantly reduces the recombination rates at *pTET-lys2-GTOP* reporter construct in Top1 mutant-expressing strains (**Figure 3C-E**). As seen with *NSR1-*deletion, the level of decrease in recombination rates due to Nsr1ΔRGG in yTop1S733E-expressing cells resembles that seen in *top1*Δ background. However, in yTop1Y727F- and yTop1Y740Stop-expressing cells, the rates of recombination at the *pTET-lys2-GTOP* reporter were significantly higher than in WT background even after deleting the RGG domain. Altogether, the *NSR1-*deletion and *Nsr1*Δ*RGG* fluctuation data alludes to two possible models explaining how expression of both Top1 catalytic mutants and Nsr1 leads to severely heightened recombination at *pTET-lys2-GTOP.* One model is that the high level of G4-associated recombination in yeast cells expressing Top1 cleavage-defective mutants is the result of an additive effect where G4s are more frequently bound by either Nsr1 or Top1 mutants. Alternatively, Top1 mutants and Nsr1 interacting and cooperatively binding to G4s could result in a synergistic effect.

**Figure 6:**
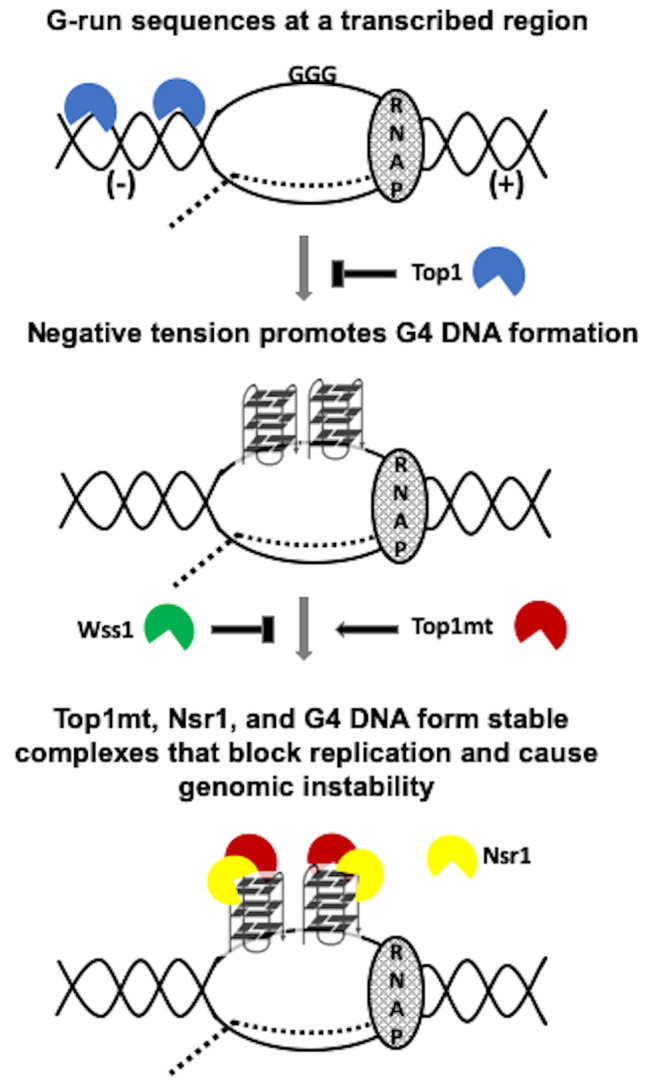
FIGURE 6: Model of co-transcriptional G4-formation and the effect of Top1 activity and mutation on G4-induced genomic instability. RNAP = RNA polymerase complex. Dotted line = the nascent transcript. (-) = negative tension behind the transcription complex. (+) = positive tension ahead of the transcription complex. Top1mt = Top1 mutant. N = Nsr1 N-term. C = Nsr1 C-term. Top1mt capable of G4-binding (i.e. Top1Y727F or Top1Y740Stop) but not Top1mt incapable of G4-binding (i.e. Top1S733E) form Top1mt/Nsr1/G4 DNA complexes that block replication and cause genomic instability.

While the exact biological relevance of the Top1/Nsr1 interaction is not completely understood, it is thought to be related to the localization of Top1 to the nucleolus [38]. Since ribosomal DNA (rDNA) located within the nucleolus is highly transcribed and Top1 relieves transcriptional helical stress, it is conceivable that Nsr1 recruits Top1 to the rDNA locus to maintain optimal helical torsion. We confirmed through co-IP experiments that WT yTop1 and Nsr1 interact (**Figure 4E**). In addition, we found that neither Top1 mutation nor deletion of Nsr1's RGG domain affects Top1/Nsr1 interaction (**Figure 4F**). This co-IP data suggests the effect of Top1 catalytic mutants and Nsr1 on G4-induced recombination could be synergistic since Top1 mutants and Nsr1 interact and we previously showed that Nsr1 is significantly enriched at *SμG4 GTOP* relative to non-G4 loci in ChIP experiments [19]. We also showed that only the expression of full-length Nsr1, but not the Nsr1 C-term or N-term (required for interaction with Top1[38]), increased the recombination rate at the *pTET-lys2-GTOP* reporter relative to the vector control in *TOP1Y727F nsr1*Δ and *TOP1Y740STOP nsr1*Δ cells (**Figure 4C** and **4D**). This indicates that the synergistic effect on G4-induced recombination requires both interaction between Nsr1 and Top1 and the interaction between Nsr1 and G4 DNA. Altogether, our data suggest a model in which Top1 catalytic mutants and Nsr1 bind to and interact on co-transcriptionally formed G4s to form a highly mutagenic complex that prevents G4-resolution and potentially impedes replication fork movement through G4-motifs (**Figure 6**). Future experiments will focus on uncovering if replication through G4-motifs is disrupted in Top1 catalytic mutant cells expressing full-length Nsr1 to better elucidate this mutagenic mechanism.

TOP1 mutations typically occurred in types of tumors that are intrinsically hypermutated, and in these tumors TOP1 mutations further exacerbated genomic instability several folds. Our use of G4 PONDS acted as a test case to assess the impact of mutated TOP1 in causing mutations at non-B DNA structures. G4 PONDS separated TOP1 mutants into two distinctive groups with respect to mutagenesis; those with mutations at the carboxy terminus and those with mutations in the rest of the protein. Mutations at the carboxy terminus, which contains residues of essential importance for its catalytic function as well as residues necessary for tight interaction with DNA, appear to exert a significantly greater impact on mutations at G4 PONDS than those in the rest of the protein (**Figure 5**). Considering that there are hundreds of thousands of PONDS genome-wide, these sites may serve as a reservoir upon which mutated TOP1 acts to elicit strong genomic instability, thereby driving the mutational burden of affected tumors.

In summary, we have found that expression of Top1 mutants, some of which are found in CPT-resistant cancer cells, sharply increases the genomic instability associated with co-transcriptionally-formed G4s in yeast. A model of genome instability at G4 DNA exacerbated by the cleavage-defective Top1 mutants is shown in **Figure 6**. While co-transcriptionally-formed negative supercoils accumulate in the absence of functional Top1 due to either the complete loss of Top1-encoding gene or mutations leading to defects in DNA binding or DNA cleavage, the G4-binding and -stabilization by the cleaveage-defective Top1 mutants further enhances the instability and recombination occurring at G4-forming genomic sites. We also discovered a new role of Wss1 in suppressing G4-associated genomic instability in presence of Top1 cleavage-defective mutants, putatively by removing Top1 mutants trapped on co-transcriptionally formed G4s. Another important finding is that the instability at G4 DNA is exacerbated by the interaction between yeast-nucleolin (Nsr1) and Top1 mutants. The findings reported here are clinically relevant since Top1 mutants arise in cancer cells in response to treatment with CPT or CPT-derivatives [67] and human nucleolin is frequently overexpressed or mis-regulated in cancer cells [57–59]. The clinical relevance is further underscored by our own finding that mutations in Top1 correlate with high mutation frequencies throughout the genome, and that mutations in the catalytic carboxy terminal domain of Top1 correlate with enrichment of mutations at G4 PONDS. Overall, our results suggest that the expression of Top1 mutants could induce additional genome rearrangements in cancer cells by supporting G4-formation and -stabilization. The resulting genomic rearrangements originating at G4-motifs may lead to secondary cancer development greatly complicating patient treatment. Other studies have documented secondary cancer development in patients following treatment with CPT-derivatives [68, 69]. In the future, it will be valuable to explore how CPT-treatment and subsequent emergence of Top1 mutants can lead to further genome instability and potential secondary cancers.

## MATERIALS AND METHODS

### Yeast strain and plasmid construction

Yeast strains used in this study were derived from YPH45 (*MATa, ura3-52 ade2-101 trp1*Δ*1*) and the construction of the *pTET-lys2-GTOP* or *–GBTM* reporter containing strains was previously described [23]. Unless noted otherwise, all yeast gene knock out and Top1 and Nsr1 epitope-tagged strains were constructed by one-step allele replacement where parental strains were transformed by the LiOAc method [70] with PCR products containing selectable marker cassettes. All PCR primers used in strain construction and allele replacement are listed in Table S1. All tagged and mutated strains were confirmed by Sanger sequencing. The construction of *yTOP1Y727-3XFLAG* strains was previously described [28]. The *yTOPY740Stop-3XFLAG* mutant strains were constructed using the Top1Y740Stop F and Top1Y740Stop R primers. The *yTOP1S733E-3XFLAG* mutant strains were created using the “delitto perfetto” (funny, it means perfect murder) method [71]; *yTOP1-3XFLAG* strains were first transformed with PCR product created with the Top1S733E F and Top1S733E R primers to insert the *URA3* marker near the S733 codon. A second transformation with a duplex oligo containing the S733E mutant codon (primers Top1S733E and Top1S733E RC annealed) was performed, resulting in the loss of the *URA3* marker and 5-Fluoroorotic acid resistance. The *NSR1-6XHA* strains were constructed by transformation with PCR product consisting of the pHYG-AID*-6HA plasmid [72] amplified with the Nsr1-6XHA F and Nsr1-6XHA R primers. The *Nsr1*Δ*RGG-6XHA* strains were constructed by transformation with PCR product consisting of the pHYG-AID*-6HA plasmid amplified with the Nsr1ΔRGG-6XHA F and Nsr1ΔRGG-6XHA R primers.

The pADH1-Nterm Nsr1 (N-term Nsr1; amino acids 1-171) and pADH1-Cterm Nsr1 (C-term Nsr1; amino acids 172-414) plasmids were gifts from A. Saiardi from the University College London, UK [39]. The pADH1-Nsr1 (Nsr1 FL; amino acids 1-414) plasmid was constructed by replacing the Nsr1 N-terminus DNA sequence from pADH1-Nterm Nsr1 with the full length Nsr1 open reading frame that was PCR-amplified from the pRS316-derived Nsr1-expression *CEN* vector described in [19].

### Western blotting

Yeast whole cell lysates were prepared for western blotting as previously described [73]. Whole cell lysate samples were centrifuged and resuspended in 2X SDS sample buffer and boiled for 10 min at 95 °C before being resolved on 4-20% SDS-PAGE gels (Bio-Rad; Cat# 456-1093). Proteins were transferred to a PVDF membrane using a Trans-Blot® SD cell machine (Bio-Rad; Cat# 170-3940) and then probed with either an α-FLAG antibody conjugated to horse radish peroxidase (HRP) (Sigma; Cat# A8592), an HRP-conjugated α-HA antibody (Sigma; Cat# H6533), an α-HRP-conjugated GAPDH antibody (Invitrogen; Cat# MA5-15738-HRP), or a primary α-GST antibody (Invitrogen; Cat# MA4-004) followed by incubation with a secondary HRP-conjugated α-mouse Ig antibody (R&D Systems; Cat# HAF007) as indicated in the respective figures. Blots were visualized by treatment with GenDEPOT West-Q Femto ECL (Cat# W3680-010) and a Bio-Rad ChemiDoc™ MP imaging system. Quantification of Top1 and Nsr1 protein levels was performed using Image Lab software. The pixel volumes of FLAG- or GST-bands after subtracting background were divided by GAPDH loading control pixel volumes. Averages and standard deviations from at least three independent experiments were calculated and a student's T-test (GraphPad Prism) was used to assess statistical differences where indicated.

### Determination of recombination and mutation rates

Fluctuation analysis was performed as previously described [23]. Briefly, 12-36 individual 1 mL cultures of each strain were used for fluctuation analyses and the recombination rates were calculated using the method of median as described previously [74]. Recombination rates are considered significantly different if their 95% confidence intervals indicated with error bars do not overlap. For fluctuation analysis of *NSR1-*deletion strains expressing different Nsr1 protein constructs, cells were transformed with pADH1-Nterm Nsr1, pADH1-Cterm Nsr1, pADH1-Nsr1, or pRS426 as a vector control. Twelve individual colonies per strain were used to inoculate 1 mL cultures in synthetic complete media lacking uracil and containing 2% glucose (SCD-Ura) and grown at 30°C. After 4 days, cultures were washed and diluted appropriately and then plated on agar plates containing either SCD-Ura for determination of total CFU or SCD-Ura/-Lys for determination of Lys+ recombinants. Recombination rates were determined as described above. Where applicable, doxycycline hyclate (Sigma) was added to the growth media to the concentration of 2 μg/ml.

### *In vitro* DNA binding assays

*In vitro* DNA binding assays were performed as previously described with some modifications [75]. Oligos with 5'- and 3'-biotin attachments used in pull downs were purchased from Sigma. For each sample, 25 pmol of biotinylated oligos were folded by boiling for 5 min at 95°C in a heat block followed by slow cooling to room temperature in 100 μL of 10 mM Tris pH 7.5 + 100 mM KCl. Folded oligos were incubated while rotating with 6.25 μL of Streptavidin-Coupled M-280 Dynabeads (Invitrogen; Cat# 11205D) that were washed twice and resuspended in 10 mM Tris HCl pH 7.5 + 1 mM EDTA + 300 mM KCl. After 1 h incubation at room temperature, oligo-bound beads were washed twice with and resuspended in 5 mM Tris HCl pH 7.5 + 0.5 mM EDTA + 150 mM KCl and kept at 4°C until further use. Oligo-bound beads were rinsed once with 1 mL lysis buffer B (50 mM Hepes pH 7.5, 1 mM EDTA pH 8.0, 300 mM KCL, 10% glycerol, 0.05% NP40, 1mM PMSF, 1 mM DTT, and fungal protease inhibitor cocktail (Sigma; Cat# P8215; 50 μl/ 10 ml)) and were resuspended in 100 μL lysis buffer B right before being added to yeast whole cell lysates.

For yeast whole cell lysate preparation, a 5 mL YPD overnight culture was used to inoculate a 500 mL YPD culture and cells were grown shaking at 30°C until the culture reached an OD_600_ of 1.5 – 2.0. A total of 410 OD_600_ of cells were collected via centrifugation at 4°C after washing twice with H_2_O and once with lysis buffer A (50 mM HEPES pH 7.5, 1 mM EDTA pH 8.0, 300 mM KCL, and 10% glycerol) followed by freezing at -80°C. Frozen pellets were resuspended in lysis buffer B and lysates were prepared by four rounds of bead beating with acid washed glass beads for 30 sec followed by 5 min of incubation on ice. After bead beating, lysates were sonicated for 10 cycles of 20 sec ON/40 sec OFF at low amplitude with a Bioruptor (Diagenode) at 4°C. Lysates were clarified by centrifugation at 4°C and oligo-conjugated streptavidin magnetic beads were added. A magnet was used to pull down beads after overnight incubation rotating at 4°C followed by four 1 mL washes in lysis buffer B and elution in 50 μL 2X SDS sample buffer. Subsequent western blot analyses of elutions and input samples (clarified lysate) were carried out as described above. Pull down/Input was calculated by dividing the pull-down pixel volumes by the input pixel volumes. Averages and standard deviations of ratios from three independent experiments were calculated and a student's T-test (GraphPad Prism) was used to assess statistical differences where indicated.

### Top1-SUMO Pull Down

pGPD2-His-SMT3 was constructed by cloning the PCR-amplified His-SMT3 sequence from the pYlplac211-ADH-His-SMT3 plasmid (a gift from Stefan Jentsch lab) into the pGPD2 vector (Addgene Cat# 43972) using BamH1 and Xma1 restriction sites. pGPD2-His-SMT3 was transformed into *wss1*Δ strains harboring the indicated *TOP1* alleles. Three to five colonies from SCD-ura selection plates containing 2% glucose were used to inoculate 5 mL of SCD-ura liquid media containing 2% glucose and were grown overnight at 30 °C. The next day, 2.5 mL of overnight culture was added to 50 mL SCD-ura liquid media containing 2% glucose and cultures were grown at 30 °C shaking until they reached an OD_600_ of ~1. Then, a total of 50 OD_600_ of cells were collected via centrifugation at 4°C and froze at -80°C after washing twice with H_2_O. The pull-down of SUMO-conjugated proteins from whole cell lysates using HisPur Ni-NTA Resin (Thermo Scientific; Cat# 88221) was performed as previously described [76]. Input and pull-down samples were resolved by SDS PAGE and transferred to PVDF membrane with a Trans-BlotSD cell machine (Bio-Rad; Cat# 170-3940). Blots were probed with an α-FLAG-HRP antibody (Sigma; Cat# A8592) and visualized by treatment with ECL substrate (GenDEPOT; Cat# W3680-010) and a Bio-Rad ChemiDoc MP imaging system.

### Co-immunoprecipitation (co-IP) experiments

For each sample, 500 μL of saturated overnight culture was added to 50 mL of fresh YEP media with 2% glucose (YEPD) and grown shaking at 30°C until they reached an OD_600_ of 1-1.5. A total of 44 OD_600_ of cells were collected by centrifugation at 4 °C and washed once in lysis buffer C (50 mM Tris pH 8, 150 mM NaCl, and 7 mM EDTA) before pellets were frozen at -80°C. Pellets were lysed by the addition of 500 μL of lysis buffer D (lysis buffer C with 5 mM DTT, 1 mM PMSF, and fungal protease inhibitor cocktail (Sigma; Cat# P8215; 50 μL/10 mL)) and acid washed glass beads followed by four rounds of bead beating for 30 sec followed by 5 min of incubation on ice. Next, lysates were clarified by centrifugation at 4°C and 500 μL of lysis buffer D was added to each sample. Fifty μL of equilibrated anti-Flag M2 Affinity Gel Beads (Sigma; Cat# A2220) were added to each lysate and samples were incubated for 1 hr rotating at 4°C. Anti-Flag M2 Affinity Gel Beads were then collected by centrifugation at 8,000 x g for 30 sec and washed four times. Each wash consisted of 1) centrifugation at 8,000 x g for 30 sec, 2) removal of supernatant, 3) addition of 1 mL wash buffer E (lysis buffer D with 0.75% Triton X-100), and then rotation at 4°C for 25 min. After the last wash, beads were resuspended in 20 μL of lysis buffer D. Proteins were eluted from the anti-Flag M2 Affinity Gel Beads by adding 20 μL of 200 μg/mL 3X-FLAG peptide (Sigma; Cat# F4799) and then incubating for 30 min at room temperature while rotating. After elution, samples were centrifuged and 30 μL of supernatant was collected and placed into a fresh 1.5 mL epitube. Fifteen μL of 4X SDS sample buffer was added to the collected supernatants and samples were boiled for 10 min at 95°C before being resolved on a 4-20% SDS PAGE gel (Bio-Rad; Cat# 4561093) and subjected to western blotting. Quantitative and statistical analyses of the co-IP were carried out as indicated for the oligo pull-down procedure above.

### Mutations in cancer genomes

Mutations in cancer were obtained from the Catalogue of Somatic Mutations In Cancer (COSMIC) at https://cancer.sanger.ac.uk/cosmic through file CosmicMutantExport_v94.tsv. We used custom scripts to generate a child file containing only genome-wide sequencing data, to which we added the total number of mutations per sample. For samples with mutations in *TOP1*, we chose mutations resulting in single non-synonymous substitutions, except for termination codons which were from either nonsense mutations or short frameshifts. Samples with mutations in TOP1 were then divided in five groups according to the domain where they occurred: Stop (frameshifts and/or nonsense mutations anywhere in the coding region), N-Ter (missense mutations in a.a. 1 – 214), Core (missense mutations in a.a. 215 – 634), Linker (missense mutations in a.a. 635 – 711), and C-Ter (missense mutations in a.a. 712 – 765) [77]. For Core, we removed 4 samples (2, 61, 7C and HCC38), which in the original COSMIC dataset were associated with more than one types of tumor.

The coordinates for the G4 potential non-B DNA-forming sequences (PONDS) [12] were obtained with a custom C++ program that ran in a parallel environment using the Message Passing Interface (MPI) using the reference human genome assembly GRCh38/hg38. The script is available at https://github.com/abacolla. Intersections between the genomic coordinates of mutations and G4 PONDS were computed with custom C++ scripts run in parallel.

## SUPPLEMENTAL MATERIAL

Click here for supplemental data file.

All supplemental data for this article are available online at https://www.microbialcell.com/researcharticles/2021a-berroyer-microbial-cell/.

## Abbreviations:

ChIP-seq: – chromatin immunoprecipitation sequencing,
CPT: – camptothecin,
DPC: – DNA–protein cross-link,
G4: – guanine quadruplex,
PONDS: – potential non-B DNA-forming sequences,
rNMP: – ribonucleoside monophosphate,
Top 1: – topoisomerase 1,
WT: – wild type.
