# Supplementary material for: Cleavage-defective Topoisomerase I mutants sharply increase G-quadruplex-associated genomic instability
Source: Microb Cell. 2022 Jan 31;9(3):52–68. doi: 10.15698/mic2022.03.771 (PMC8890623; doi:10.15698/mic2022.03.771)
Supplement: Supplementary file 1 [file mic-09-052-s01.pdf]

Supplementary Tables

Table S1. Primers used for strain construction in this study.

Primer	Sequence (5' to 3')
Top1Y740Stop F	CCAGGTTTCACTGGGCACTTCCAAAATCAATTATATAGACCCTAGACTTTCTGTGGTATTTTGCAAAAAGAGGGAACAAAAGCTGGAG
Top1Y740Stop R	CCGATTCTATGGCCCATTTGAATTTTTCTCTTAGGGTTTTTGTAAAAATCTTTTCAATCGGAACATCATACTATAGGGCGAATTGGGT
Top1S733E F (Allele replacement step 1)	GTTGAAAGATAAAGAGGAAAACTCCCAGGTTTCACTGGGCACTTCCAAAATCAATTATATAGACCCTAGACTTTCTCAGCTGAAGCTTCGTACG
Top1S733E R (Allele replacement step 1)	GAATTTTTCTCTTAGGGTTTTTGTAAAAATCTTTTCAATCGGAACATCATACTTTTTGCAAAATACCACAGGCCACTAGTGGATCTG
Top1S733E (Allele replacement step 2)	GGAAAACTCCCAGGTTTCACTGGGCACTTCCAAAATCAATTATATAGACCCTAGACTTGAAGTGGTATTTTGCAAAAAGTATGATGTTCCGATTGAAAAGATTTTTACAAAAACCCTAAG
Top1S733E RC (Allele replacement step 2)	CTTAGGGTTTTTGTAAAAATCTTTTCAATCGGAACATCATACTTTTTGCAAAATACCACTTCAAGTCTAGGGTCTATATAATTGATTTTGGAAGTGCCCAGTGAAACCTGGGAGTTTTCC
Nsr1-6XHA F	TGCTAATACTGCTCCATTGGGCAGATCAAGAAATACCGCTTCTTTCGCTGGTTCAAAGAAAACATTTGATCCCGGGAAATACCCATAC
Nsr1-6XHA R	TCAAAAATGGAATATAGTAATTAACGTAAAAAGAGAAAAAATTGAAATTGAAATTCATTTCATTTTCTCAGTTAAAGCCTTCGAGCGTCCC
Nsr1ΔRGG-6XHA F	GGTGAATACATTGACAACAGACCAGTTAGATTAGACTTCTCTTCTCCAAGACCAAACAACGATCCCGGGAAATACCCATAC
Nsr1ΔRGG-6XHA R	AATGGAATATAGTAATTAACGTAAAAAGAGAAAAAATTGAAATTGAAATTCATTTCATTTTCTCAGTTAAAGCCTTCGAGCGTCCC

Table S2. Catalytic activity of Top1 mutants determined by measuring the Lys+ mutation rate of yeast strains harboring a *pTET-lys2-AG4* reporter.

**Strain**	**Lys+ Mutation Rate x 10^{-8} (CI)**
WT rnh201Δ	1980 (1860-2250)
top1Δ rnh201Δ	9.21 (7.52-13.7)
TOP1Y727F rnh201Δ	13.1 (10.2-17.5)
TOP1Y740STOP rnh201Δ	10.9 (8.30-13.9)
TOP1S733E rnh201Δ	10.7 (9.23-15.8)

Rates are considered statistically significantly different if their 95% confidence intervals do not overlap [74]. CI = 95% confidence interval.

Table S3 – S13 contain raw data for Figure 5 and linked as metadata.

Table S14. P-values for Figure 5B.

**Group A**	**Group B**	**p-values (Wilcoxon Test)**
Ran	RanH	0
N-Ter+Linker+Core	Ran	0
Stop+C-Ter	Ran	2.75E-14
Stop+C-Ter	RanH	0.3678
N-Ter+Linker+Core	RanH	0.3942
N-Ter+Linker+Core	Stop+C-Ter	0.7719

P-values were from Wilcoxon tests. For the purpose of single Wilcoxon tests, we combined the numbers of mutations and numbers of samples when applicable.

Table S15. P-values for Figure 5C.

**Group A**	**Group B**	**p-values (Wilcoxon Test)**
Ran	RanH	1.11E-15
N-Ter+Linker+Core	Ran	0.008031
Stop+C-Ter	Ran	3.03E-4
Stop+C-Ter	RanH	0.6555
N-Ter+Linker+Core	RanH	3.25E-5
N-Ter+Linker+Core	Stop+C-Ter	0.0184

P-values from Wilcoxon tests. For the purpose of single Wilcoxon tests, we combined the percent mutations at G4 and numbers of samples when applicable.

Supplemental Figures

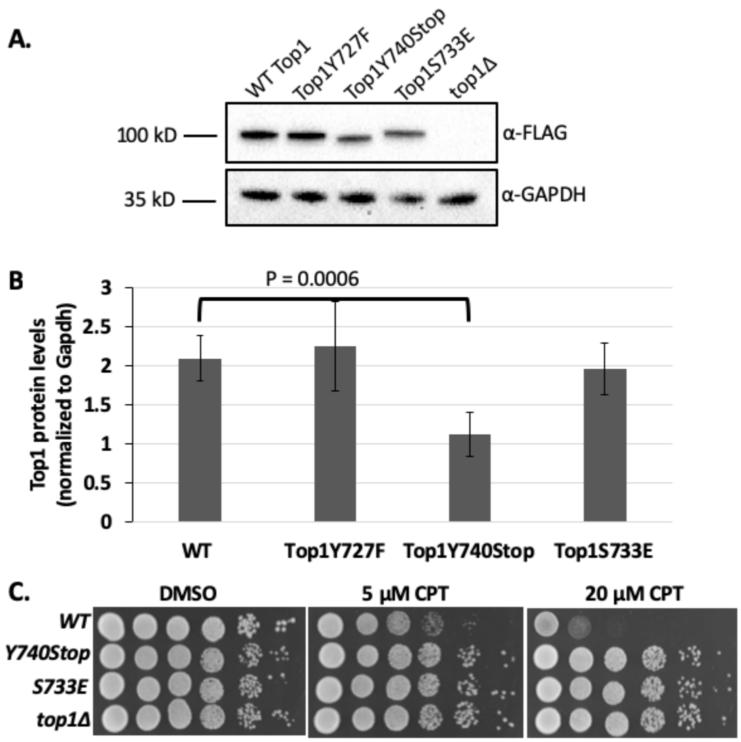

Figure S1. Yeast homologs of human Top1 mutants found in cancer cells are expressed. *A.* Western blot of cell extracts prepared from yeast strains expressing FLAG-tagged Top1 proteins. Blots were probed with αFLAG and αGAPDH antibodies. *B*. Quantification of Top1 mutant protein levels. Means and standard deviations of Top1 pixel intensities normalized to Gapdh pixel intensities from 5 western blots performed as in A. Significance of statistical differences was determined by Student T-tests (GraphPad Prism). *C*. Yeast Top1 mutants Y740Stop and S733E are resistant to CPT. Spot assays of *MUS81*-deletion yeast cells on control plates (YPD and DMSO) or plates containing the indicated concentrations of CPT. Cells were diluted 1:10 serially before spotting on plates. Images shown were taken 2 days after growth at 30 °C.

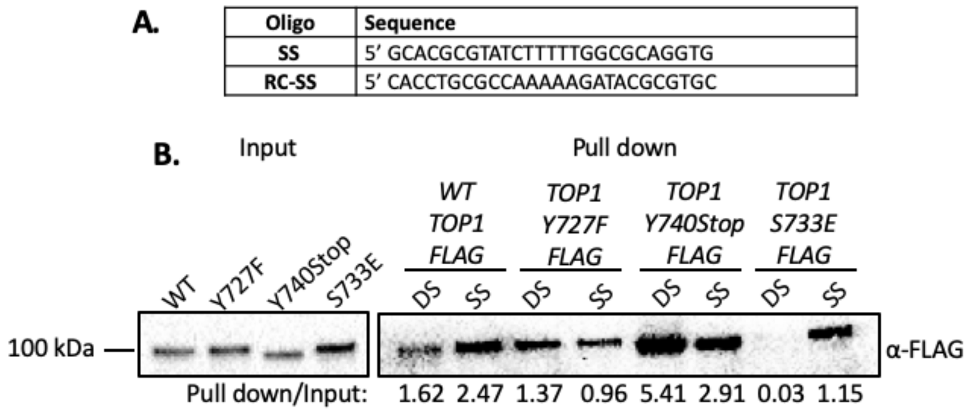

Figure S2. Top1 catalytic mutants bind duplex DNA substrates *in vivo*, while Top1 DNA binding mutants do not. *A.* Sequences of oligonucleotides (oligos) used in *in vitro* binding oligo binding assay shown in B. *B.* Western blot of *in vitro* binding assay utilizing biotinylated oligos conjugated to streptavidin magnetic beads and yeast whole cell lysates prepared from cells expressing FLAG-tagged Top1 proteins. Blot was probed with an α-FLAG-HRP (Sigma) antibody. SS = biotinylated single-stranded oligo control. DS = SS biotinylated oligo annealed to non-biotinylated RC oligo to create a duplex substrate. Quantification of binding is below blot and was calculated by dividing Input pixel intensities from Pull down pixel intensities.

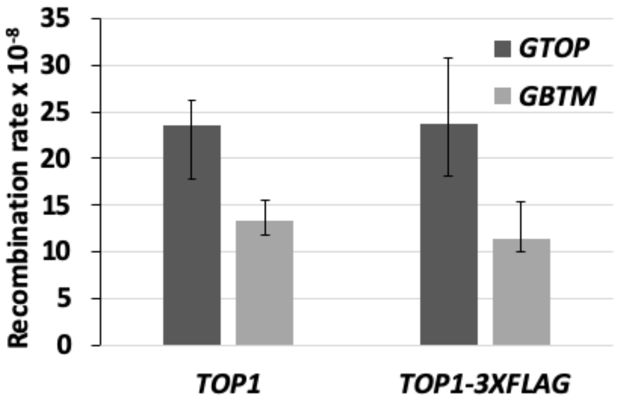

Figure S3. C-terminal 3X-FLAG tag does not impact the function of WT yTop1. Recombination rates of WT yTop1 tagged and untagged recombination reporter strains. *GTOP* indicates recombination at G4-motif while *GBTM* indicates recombination at a loci not prone for G4-formation. Rates are considered statistically significantly different if their 95% confidence intervals (shown as error bars) do not overlap [74].

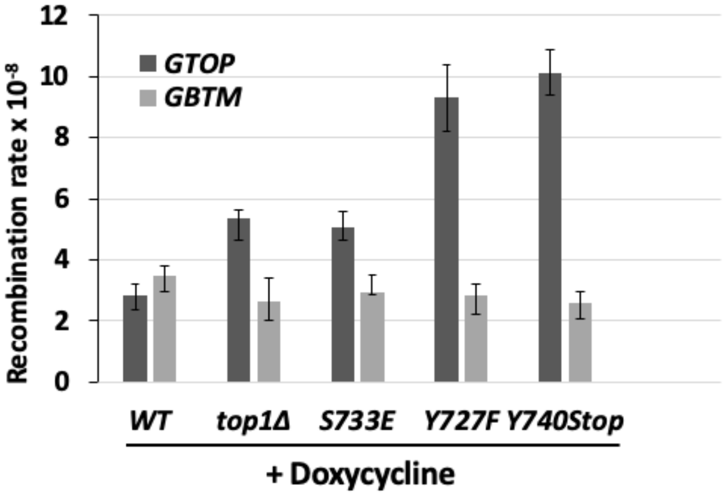

Figure S4. The effect of transcriptional repression on the *GTOP* and *GBTM* recombination rates of Top1 mutant strains. Doxycycline was added to the media of fluctuation cultures to repress transcription from the *pTET* promoter upstream of the *SμG4 GTOP* and *GBTM* constructs. Rates are considered statistically significantly different if their 95% confidence intervals (shown as error bars) do not overlap [74].

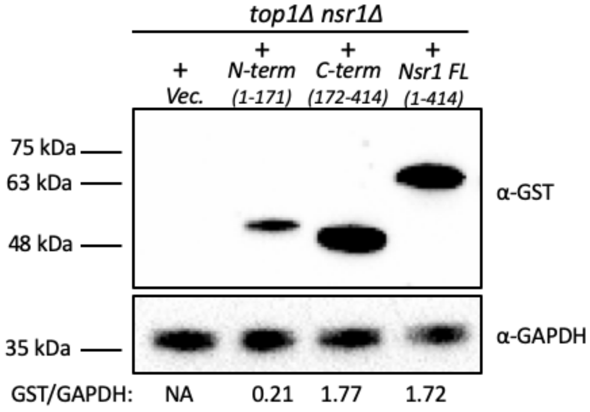

Figure S5. Nsr1 constructs are expressed from pADH1-Nterm Nsr1, pADH1-Cterm Nsr1, and pADH1-Nsr1 in yeast. Western blot of lysates prepared from *top1*Δ *nsr1*Δ cells transformed with control, pADH1-Nterm Nsr1, pADH1-Cterm Nsr1, and pADH1-Nsr1 plasmids. Nsr1-GST construct blots were probed with a primary α-GST antibody (Invitrogen) and a secondary α-mouse IgG-HRP antibody (R&D Systems-biotechne). Loading control blots were probed with an α-GAPDH antibody (Invitrogen). N-term = pADH1-Nterm Nsr1, C-term = pADH1-Cterm Nsr1, Nsr1 FL = pADH1-Nsr1, and Vec. = vector control. Quantifications of Nsr1 construct protein levels are listed below blot.

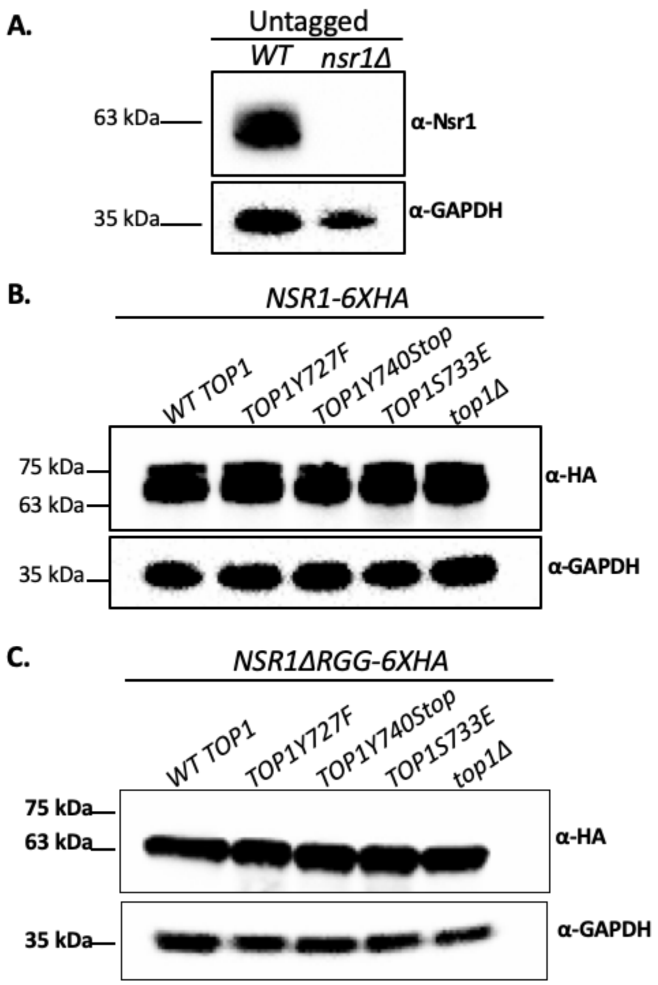

Figure S6. *NSR1-6XHA* and *NSR1ΔRGG-6XHA* proteins are expressed in Top1 mutant backgrounds. *A.* Western blot of lysates prepared from yeast cells expressing untagged, endogenous Nsr1 or *nsr1Δ* yeast cells. Untagged Nsr1 blot was probed with a primary α-Nsr1 antibody (Invitrogen) and a secondary α-mouse IgG-HRP antibody (R&D Systems-biotechne). *B-C***.** Western blot of lysates prepared from Top1 mutant cells expressing either HA-tagged full length Nsr1 or HA-tagged Nsr1ΔRGG. HA-tagged Nsr1 and Nsr1ΔRGG blots were probed with an α-HA-HRP antibody (Sigma). Loading control blots in A.-C. were probed with an α-GAPDH antibody (Invitrogen).

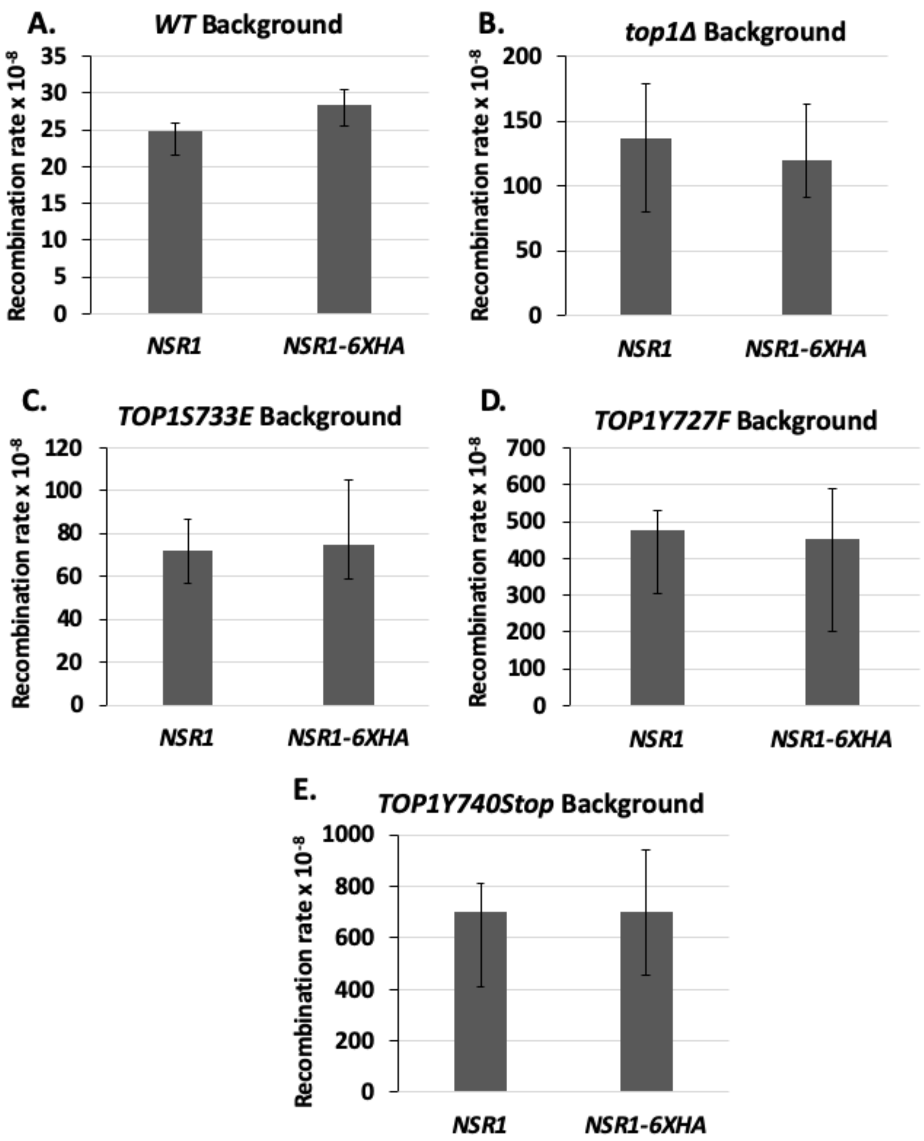

Figure S7. *NSR1-6XHA GTOP* strains and untagged *NSR1 GTOP* strains have comparable G4-induced recombination rates *A.-E.* Recombination rates of Top1 mutant *NSR1-6XHA GTOP* strains shown with the rates of respective parental strains. Rates are considered statistically significantly different if their 95% confidence intervals (shown as error bars) do not overlap [74].

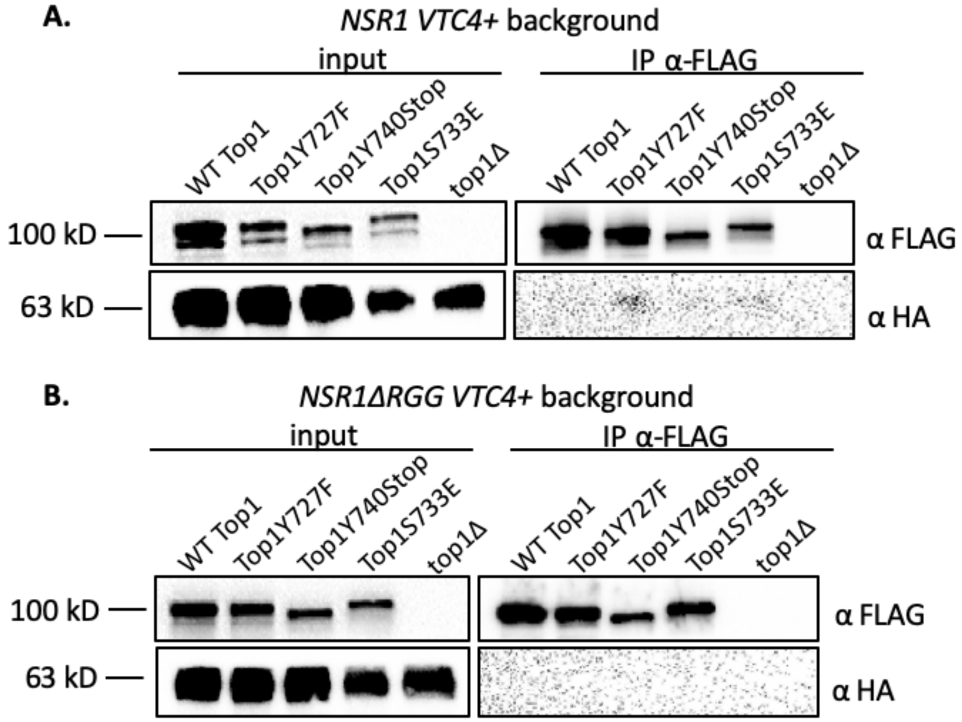

Figure S8. Top1 mutant and Nsr1 co-immunoprecipitation (co-IP) experiments in the presence of *VTC4*. *A.* Co-IP experiments conducted with *VTC4*+ yeast strains expressing Top1 proteins that are C-terminally tagged with 3X-FLAG and full-length Nsr1 that is C-terminally tagged with 6X-HA. α-FLAG coated agarose beads (Sigma) were incubated with yeast whole cell lysates. After pull down and washing, proteins were eluted from the beads by incubation with 3X-FLAG peptide (Sigma) and samples were ran on an SDS PAGE followed by western blotting. Blots were probed with either α-FLAG-HRP (Sigma) or α-HA-HRP (Sigma) antibodies. *B.* Co-IP experiments conducted with *VTC4*+ yeast strains expressing Top1 proteins that are C-terminally tagged with 3X-FLAG and Nsr1ΔRGG that is C-terminally tagged with 6X-HA. Co-IP and western blotting was performed as in A.

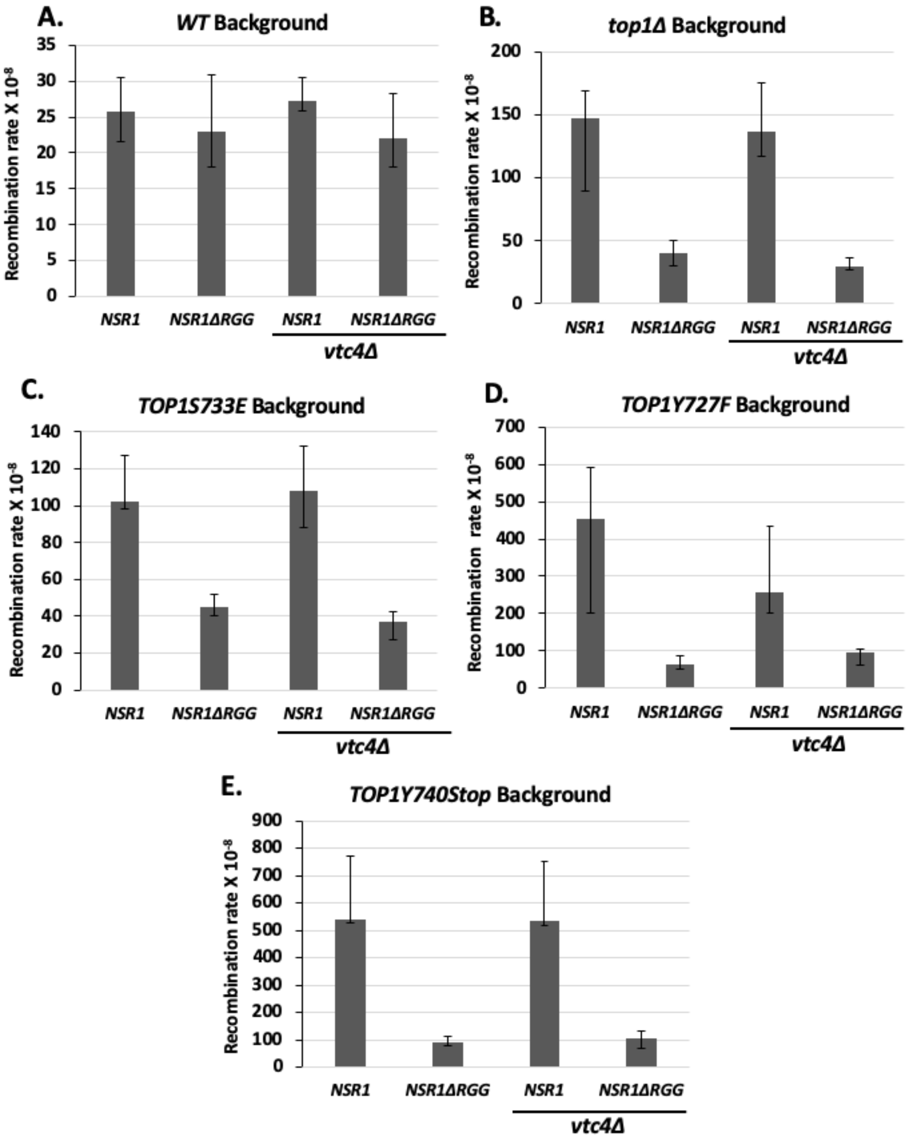

Figure S9. Deletion of *VTC4* does not affect G4-induced recombination in Top1 mutant strains. *A.-E.* Recombination rates of Top1 mutant *VTC4+* and *vtc4*Δ *GTOP* yeast strains. Rates are considered statistically significantly different if their 95% confidence intervals (shown as error bars) do not overlap [74].

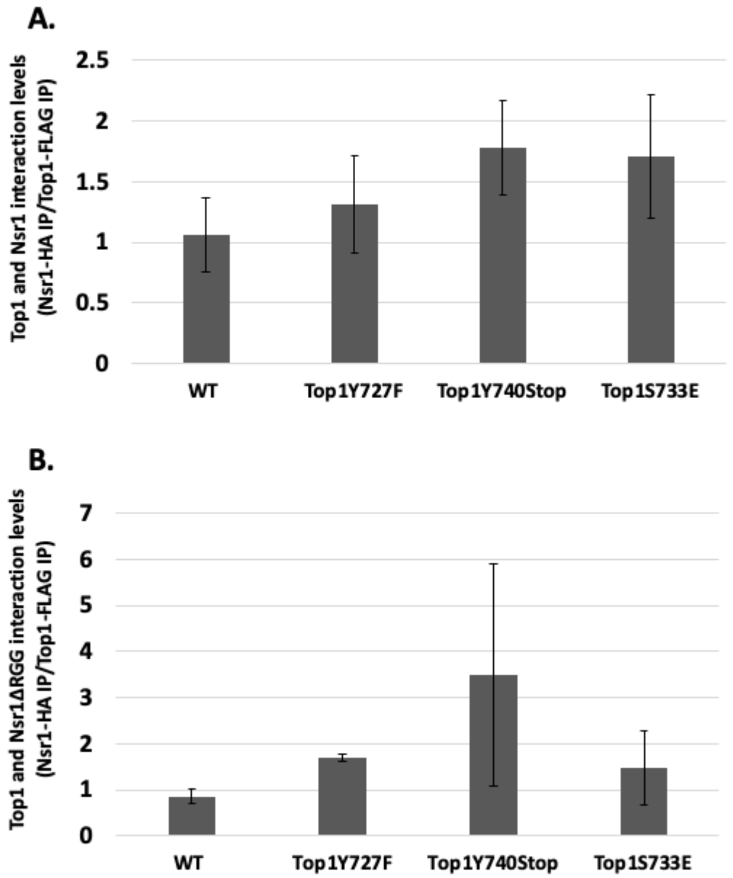

Figure S10. Quantification of Top1 mutant interactions with Nsr1. *A.* Quantification (means and standard deviations of Nsr1-6XHA IP pixel intensities normalized to Top1-3XFLAG pixel intensities) of western blots from 3 co-immunoprecipitation (co-IP) experiments performed as in Figure 4E with yeast cells expressing FLAG-tagged Top1 proteins and HA-tagged full length Nsr1*. B.* Quantification (means and standard deviations of Nsr1ΔRGG-6XHA IP pixel intensities normalized to Top1-3XFLAG pixel intensities) of western blots from 3 co-IP experiments performed as in Figure 4F with yeast cells expressing FLAG-tagged Top1 proteins and HA-tagged Nsr1ΔRGG*.*

Supplemental Methods and Materials

CPT Spot assay materials and methods.

MUS81-deletion cells were grown in 5 mls of liquid YPD media overnight at 30°C shaking. The next day, cultures were diluted to an OD_{600} of 0.15 in 5 mls of liquid YPD media and were grown at 30°C until the cultures reached an OD_{600} of 0.6-0.7. A total of 0.3 OD_{600} of cells were collected from each culture and were resuspended in 100 µl of sterile water. Then, 5 µl of serial 1:10 dilutions of cells were spotted onto YPD plates containing either 0.35% DMSO, 5 µM CPT + 0.35% DMSO, or 20 µM CPT + 0.35% DMSO. Spot assay plates were imaged every day for 2 days after growth at 30 °C using a Bio-Rad ChemiDoc™ MP imaging system. CPT was purchased from Sigma (Cat# 208925).
